# Identifying volatile organic compounds used for olfactory navigation by homing pigeons

**DOI:** 10.1038/s41598-020-72525-2

**Published:** 2020-09-28

**Authors:** Nora Zannoni, Martin Wikelski, Anna Gagliardo, Atif Raza, Stefan Kramer, Chiara Seghetti, Nijing Wang, Achim Edtbauer, Jonathan Williams

**Affiliations:** 1 Department of Atmospheric Chemistry, Max Planck Institute for Chemistry, Hahn-Meitner weg 1, 55128 Mainz, Germany; 2 Department of Migration, Max Planck Institute of Animal Behavior, Radolfzell, Germany; 3 Centre for the Advanced Study of Collective Behaviour, University of Konstanz, Konstanz, Germany; 4 Department of Biology, University of Pisa, Pisa, Italy; 5 Department of Computer Sciences, Johannes Gutenberg University, Mainz, Germany

**Keywords:** Animal behaviour, Environmental chemistry, Animal migration, Analytical chemistry, Atmospheric science

## Abstract

Many bird species have the ability to navigate home after being brought to a remote, even unfamiliar location. Environmental odours have been demonstrated to be critical to homeward navigation in over 40 years of experiments, yet the chemical identity of the odours has remained unknown. In this study, we investigate potential chemical navigational cues by measuring volatile organic compounds (VOCs): at the birds’ home-loft; in selected regional forest environments; and from an aircraft at 180 m. The measurements showed clear regional, horizontal and vertical spatial gradients that can form the basis of an olfactory map for marine emissions (dimethyl sulphide, DMS), biogenic compounds (terpenoids) and anthropogenic mixed air (aromatic compounds), and temporal changes consistent with a sea-breeze system. Air masses trajectories are used to examine GPS tracks from released birds, suggesting that local DMS concentrations alter their flight directions in predictable ways. This dataset reveals multiple regional-scale real-world chemical gradients that can form the basis of an olfactory map suitable for homing pigeons.

## Introduction

That the atmospheric composition is critical to a pigeon’s ability to home was first discovered by Wallraff over 50 years ago^1^. In those experiments, two sets of pigeons were tested after being raised in aviaries with clear (glass) and obscured (louvered screen) views of the surroundings, in order to assess whether the view of the horizon was critical for the development of their navigational abilities. Contrary to the expectation, the pigeons raised in aviaries surrounded by clear glass screens displayed impaired orientation, while those with the shielded views but unimpeded inflow of air, oriented homeward^1,2^. These findings were initially explained as an undetermined “atmospheric factor”. Shortly thereafter, Papi et al.^3^ discovered that pigeons with severed olfactory nerves are unable to find their way home. In order to explain both aforementioned empirical findings, Papi proposed the olfactory navigation hypothesis^4^. This propounded that pigeons at the home loft associate wind-borne odours with wind directions to build up an olfactory map of the region around the home area. It was further suggested that when pigeons are moved to a remote release site they are able to determine the direction of displacement based on the prevalent local odours at the release site and thereby orient homewards. This hypothesis, sometimes termed “the mosaic hypothesis” inherently assumes that each remote release location has a distinct unique odour that can be identified by the pigeon from the home aviary.

The importance of olfactory stimuli in avian navigation over unfamiliar terrain has been shown in a large number of experiments on both homing pigeons^5,6^, and wild species^7–11^. Impaired navigational performance has been consistently reported following olfactory deprivation. Further strong evidence in support of olfactory navigation in birds has been provided from experiments in which the direction of the wind experienced by the pigeons was manipulated during the map-learning period. Artificial deflection (e.g., 90°) or inversion (180°) of the wind direction experienced by pigeons at the aviary produced a corresponding deflection or inversion of the displaced pigeons’ initial orientation when homing^12,13^. Despite this evidence for atmospheric chemicals being a key component in avian navigation, the odorants involved, their origin and their distributions remain unknown. Critics of the olfactory navigation hypothesis consider the temporal instability and turbulence of the atmosphere to be incompatible with navigable spatial gradients in environmental odours over hundreds of kilometers^14,15^. In response Wallraff proposed a model of an olfactory map based on gradients of at least three ratios of volatile compounds, rather than gradients of individual molecules^5^. This model was empirically supported by a field study, in which measurements of selected volatile organic compounds (VOC) were made in several concentric circles (radius 200 km) around a pigeons’ loft. These indicated that spatial gradients of the ratios of some VOC could be stable enough to provide spatial information suitable for odour-based navigation^16^. In addition, a computer simulation study showed that the initial orientation of virtual pigeons determined based on gradients of six chemical measured compounds was comparable to that observed by releasing real pigeons in the same area^17^. Further studies found that the initial orientation and homing performances of pigeons from the same colony varied according to season, with the birds displaying better performances in spring/summer than autumn/winter^18^. One possible explanation of these results is that the prevalence or intensity of atmospheric odour signals is higher at certain times. Here we provide a first test of this hypothesis and highlight a methodology that allows for a quantitative mechanistic analysis of pigeon tracks in relation to meteorology and aerial chemicals in the future.

According to the olfactory navigation hypothesis proposed by Papi^4^ and the data collected to date, the atmospheric composition and meteorology at the home site is key to the development of the pigeons’ navigational abilities. Indeed, experience has shown that 2–4 months are required for the pigeons to associate the wind direction and the wind borne odours into the spatially and temporally varying olfactory map^19,20^. By measuring the identity and concentration of the odours experienced at the home aviary in real time during the map-learning phase, candidate navigational cue molecules could be identified and the air composition as a function of compass direction can be examined in detail. In this study, we report mixing ratios of a large suite of volatile organic compounds (VOCs), including isomeric and chirally speciated biogenic VOCs measured at high frequency (> 20,000 points), with meteorological variables during 2 months of continuous measurements at the aviary during the fledgling pigeon’s map learning phase (spring 2018). This large dataset is equivalent to that used by the birds to construct the olfactory map, assuming the odours smelled by the birds are detected by the measurement systems deployed. We investigated the specific chemical composition of three sites surrounding the birds aviary with distinct vegetation types, representative of the region (summer 2017), and we determined the VOCs mixing ratios above the region by sampling from a light aircraft at the pigeon’s flight altitude (180 m-spring 2018). The diel variability, directional dependence, and the origin of the measured VOCs are discussed. Potential candidate chemical species for homing are examined based on their atmospheric lifetime. Finally, we developed a meteorological-based analytical approach, whereby air mass trajectories are used to investigate the bird’s directional and homing performance derived from GPS tracks of recent bird release experiments conducted in the region under the same meteorological conditions of VOC measurements.

## Results

### Regional observations of volatile organic compounds

**Figure 1:**
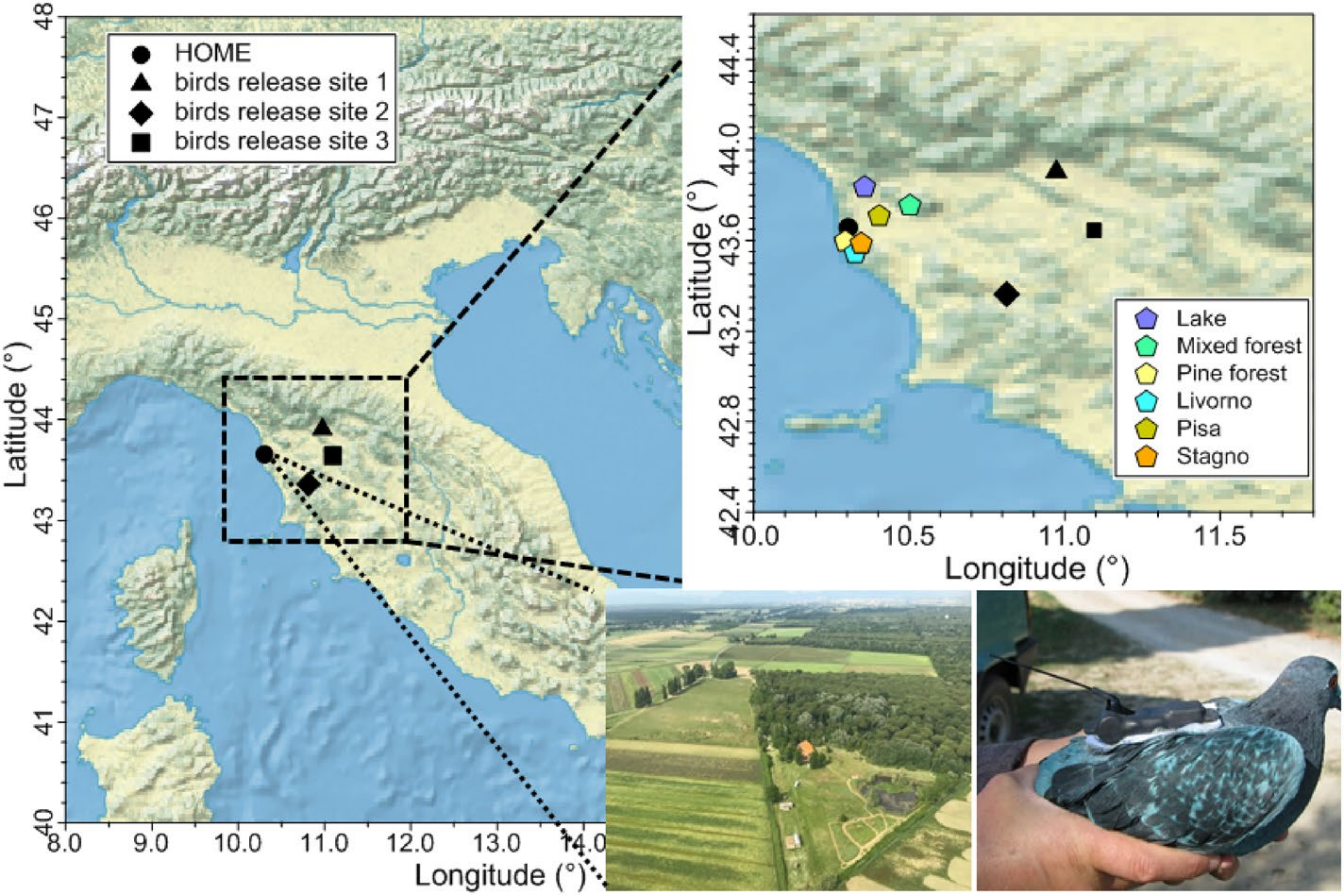
Map of the sampling area. The point designated as “home” refers to the birds aviary of Arnino (43°39′25.7″N 10°18′14.7″E) located in Tuscany in the area of Pisa-Livorno. Points on the map show the three sites used for releasing birds during a navigational experiment and the main biogenic and anthropogenic sites in the area. Photos show an aerial view of the Arnino field site during the airborne sampling (left) and a homing pigeon equipped with a GPS-tag before being released for testing its homing performance (right). Figure drawn with Igor WaveMetrics.

Measurements were conducted in summer 2017 and spring 2018 in the area of Pisa, Italy, as part of the HOMING project (Hunting Organic Molecules In NaviGation). Measurements consisted of: (i) a pilot study (summer 2017) to investigate the volatile organic compounds (VOCs) emitted by three representative local ecosystems surrounding the bird’s home aviary; (ii) a 2-month intensive field campaign (spring 2018) at the bird’s home aviary to monitor VOCs along with meteorological variables; (iii) three flights on board of a Cessna aircraft to sample at ca. 180 m, within the birds typical flight altitude (spring 2018). Specifically, (i) was conducted to identify the chemical composition of surrounding forest sites and test whether they can be smelled distinctly from the aviary. (ii) Was performed to derive the olfactory maps developed by birds based on the assumption that birds and our analytical equipment have comparable detection thresholds. (iii) Was needed to examine any regional scale gradient used by pigeons when flying. The home aviary is operated by the University of Pisa and is located at the rural site Arnino (43°39′25.7″N 10°18′14.7″E), outside the city of Pisa (11 km North–East), close to the Tyrrenian sea (1.8 km West) and the mouth of river Arno (3.2 km North) (Fig. 1).

Arnino houses hundreds homing pigeons (*Columba livia*) used for navigational experiments. Volatile organic compound mixing ratios were determined with state-of-the-art on-line and off-line analytical techniques (PTR-MS and GC–MS) and speciated in their isomeric and enantiomeric forms.

Navigational experiments entailed releasing 174 pigeons equipped with GPS loggers (N. 80/2013-A), who had never left their home before, from unfamiliar sites 50–70 km from their home (Fig. 1). For logistical reasons (power availability and shelter for technological equipment at release points), no simultaneous measurement of air composition and bird release was possible. Therefore, we used results from the most recent available flight experiments (summer 2016 and summer 2017) to determine general homing performance indicators and examined the latter in conjunction with available meteorological information, as modelled air masses trajectories.

### The daily variation of volatile organic compounds at the aviary site

**Figure 2:**
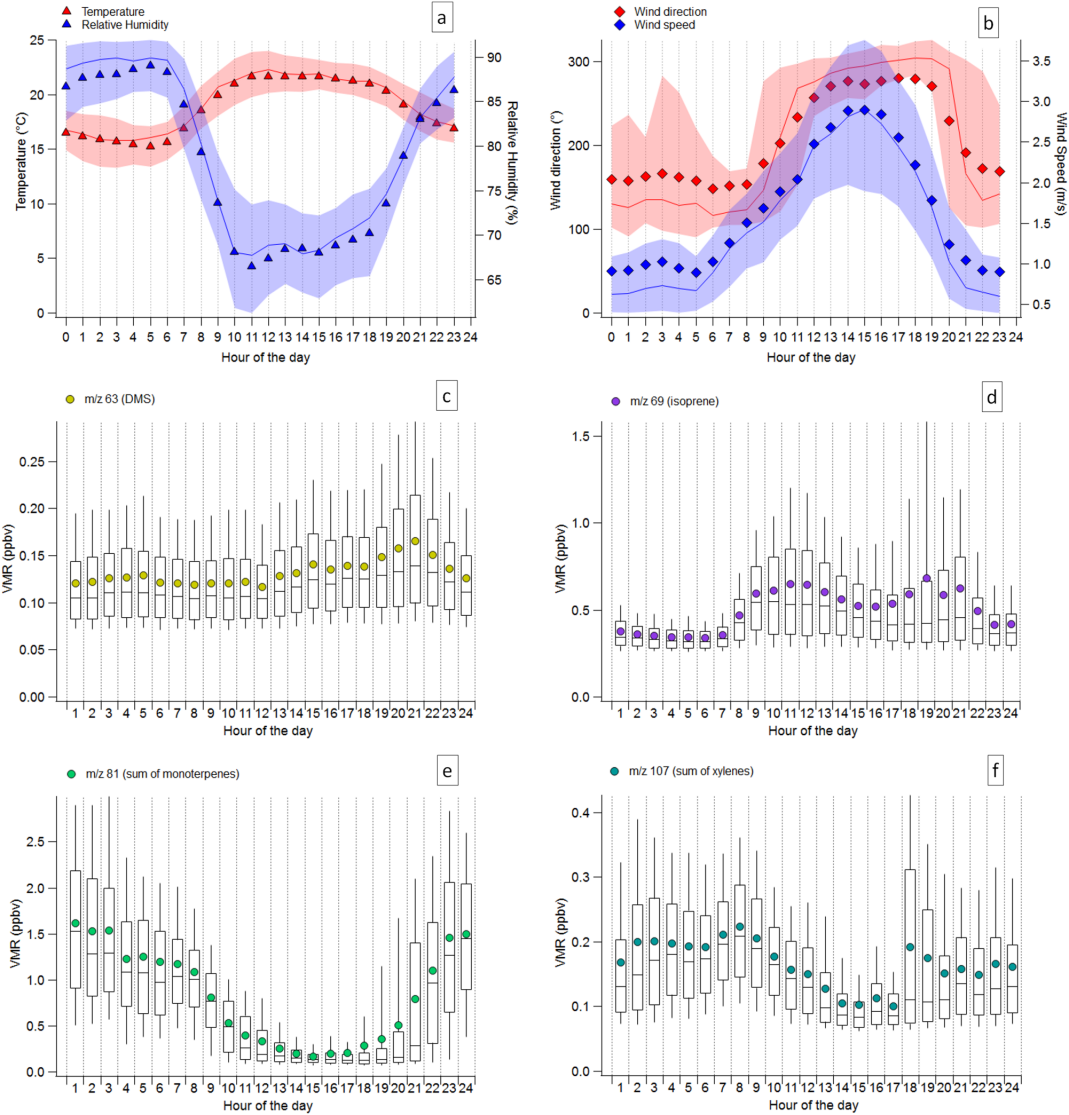
Diel cycle of meteorological parameters (**a**,**b**) and measured VOCs (**c**–**f**). The volumetric mixing ratio (VMR) of the volatile organic compounds are reported as their measured mass fragment by PTR-MS: *m/z* 63 (**c**), *m/z* 69 (**d**), *m/z* 81 (**e**) and *m/z* 107 (**f**); identified respectively as dimethyl sulphide (**c**), isoprene (**d**), sum of monoterpenes (**e**) and sum of xylenes (**f**). Meteorological parameters are plotted as mean campaign values (marker), median (line) and interquartile range (shaded area). VOC box plots report mean campaign values (marker), median (line), interquartile range (box) and 10th and 90th percentiles (whiskers).

Meteorological parameters including, temperature, relative humidity, wind direction and wind speed were measured in May–June 2018 during the ground-based campaign at the home aviary (Supplementary Fig. 1). Air temperature and relative humidity ranged between 12–25 °C and 60–85%, respectively (Fig. 2 and Supplementary Fig. 1), showing the expected mirrored diel cycle, whereby temperature is highest and RH lowest by day. Wind direction and speed exhibited a repeating daily pattern during the 2 months of measurements (Fig. 2 and Supplementary Fig. 1).

The proximity to the coast exposes the measurement site to influence by the local sea breeze system. Air masses reaching the aviary came from inland (mean wind angle 150°) every day until midday, and thereafter between 12:00 and 20:00–21:00 (local time) the air masses were advected from the sea (mean angle 270°). The wind speed, like the wind direction, also showed a diurnal profile. Figure 2 shows the diurnal profile of some compounds measured by proton transfer reaction mass spectrometry (PTR-MS). They have contrasting diel cycles and hereafter investigated further: dimethyl sulphide (DMS, *m/z* 63), isoprene (*m/z* 69), sum of monoterpenes (MT, *m/z* 81) and sum of xylenes (*m/z* 107). The full mass list and identities are reported in Supplementary Table 4 with other compound diel variations in Supplementary Fig. 2. We refer to the abbreviations included at the beginning of the manuscript for a reader not familiar with the terms used herein.

Dimethyl sulphide is a biogenic compound emitted by phytoplankton activity in seawater^21–23^; it is insoluble in water, and so readily transfers to the air at a rate dependent on the temperature and wind speed. Our measurements show that DMS mixing ratios at the site remained low and constant until 12:00, possibly reflecting weak soil emissions from the land^24^, to increase to a maximum at 20:00 due to continued marine emission, until the reversal of the wind back to offshore. The maximum hourly averaged mixing ratio of DMS is 0.17 ± 0.8 ppbv, comparable to 0.12 ppbv measured by Derstroff et al.^25^ at a coastal site on Cyprus.

Isoprene mixing ratios showed a bimodal distribution with two maxima at 11:00–12:00, and 18:00–20:00; similar to methyl ethyl ketone (MEK) and isoprene oxidation products methyl vinyl ketone, methacrolein and isoprene peroxides (MVK + MACR + ISOPOOH) and acetic acid (Supplementary Fig. 2). At both morning and afternoon maxima, the isoprene mixing ratio reached 0.7 ± 0.6 ppbv (maximum hourly average ± 1σ standard deviation). This is 10 times higher than the value observed during wintertime at a similar rural Mediterranean site in Spain^26^, but lower than the value measured in summertime by Steinbacher and coauthors^27^ at a rural site in the Po valley, Italy. Isoprene emission from terrestrial vegetation is mainly light dependent^28^, therefore near-source measurements typically exhibit a broad maximum around noon when irradiation is highest^29,30^. The two isoprene maxima observed here are the result of the sea breeze system, which brings isoprene poor marine influenced air to the site around midday.

Monoterpenes (C_10_H_16_) are emitted from vegetation, and are to a large part responsible for the scent of forests. Their sum at the site reached 1.6 ± 1 ppbv as a maximum hourly average at 01:00. Despite monoterpene emissions being also a function of temperature they do not show the same profile as isoprene. Monoterpene mixing ratios were higher at night, decreasing slowly from 03:00 and then sharply at 08:00, and remaining low through the day before rising back to early morning levels in the late evening. While isoprene is formed in the plant and released directly, mainly in response to light, many plant species emit monoterpenes day and night from resin duct storage pools contained in the leaves or needles^31^. The rates of monoterpene emission therefore depend strongly on temperature, and although lower in the night, continued emission into a shallower boundary layer can generate significant mixing ratios, as has been observed elsewhere (for example in the boreal forest^32^). As expected, air advected from the more vegetated inland had higher monoterpene mixing ratios than air from the sea. Although, marine monoterpene and isoprene emissions have been observed previously^33^, in this region terrestrial vegetation emissions clearly dominate. A similar profile (higher nighttime daytime ambient mixing ratio) was reported by Davison et al.^34^ from the Mediterranean forest of Castelporziano, however, the average daytime and nighttime concentrations were 3 times lower than reported here.

Xylenes (Fig. 2), benzene, toluene, and sum of trimethylbenzenes (Supplementary Fig. 2) are aromatic compounds predominantly emitted from anthropogenic sources, for example fossil fuel use. Mixing ratios increased in the morning at 8:00, probably due to traffic emissions, and decreased steadily between 9:00–12:00, as the wind direction changed. They remained low in the afternoon as the wind came from the sea, reflecting the expected absence of significant sources from this sector. The mixing ratios of all compounds increased again after 17:00, slowly for benzene and toluene and faster for xylenes and trimethylbenzene, suggesting different anthropogenic sources of such species (i.e. road traffic and industries). Although evidence for biogenic aromatic species has been documented^35,36^, here no evidence of natural sources of aromatic compounds was found. The maximum mixing ratios for benzene, toluene, sum of xylenes and trimethylbenzene, were 0.16 ± 0.08 ppbv, 0.32 ± 0.7 ppbv, 0.22 ± 0.09 ppbv and 0.11 ± 0.9 (hourly average maximum ± 1σ standard deviation), respectively. Similar results were found for benzene and toluene at a rural Mediterranean site in Spain (0.19 and 0.41 ppbv, respectively^26^).

### Chiral monoterpenes

**Figure 3:**
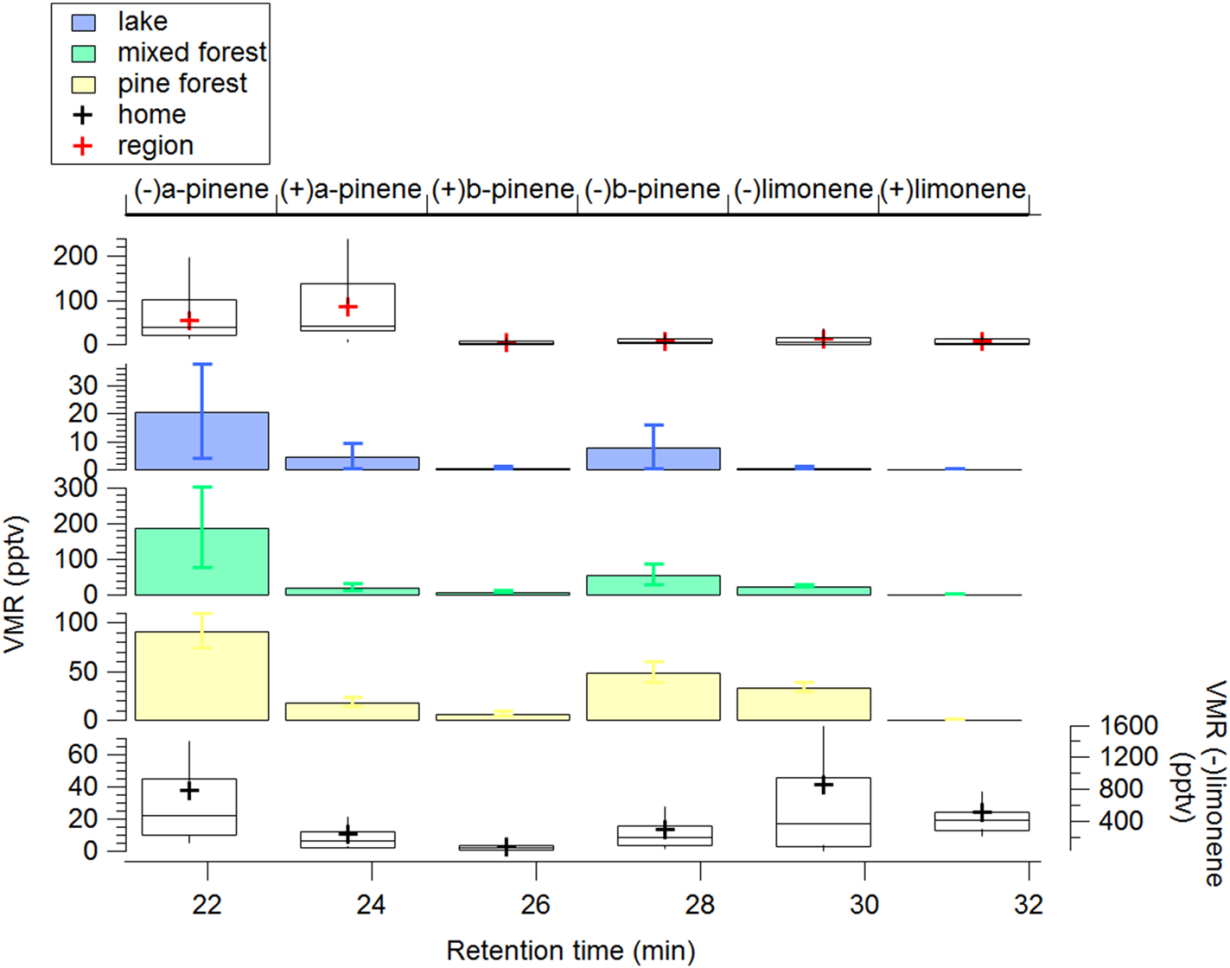
Volume mixing ratio (VMR) distribution of enantiomers from three ecosystems surrounding the birds’ aviary and at the birds’ aviary. Category plots show mean measured values with their standard deviation from three days of sampling during summer 2017. Box plots show mean campaign values (marker), median (line), interquartile range (box) and 10th and 90th percentiles (whiskers) from 2 months intensive field campaign at the birds’ aviary in spring 2018 (home) and from three flights conducted at 180 m altitude over the region during the airborne campaign. The x axes shows the retention time (min) of the chiral molecules in the gas chromatography mass spectrometer (GC–MS) and shows the good resolution achieved with the method. The left y axes indicate the volume mixing ratio of all molecules, except for (−)limonene measured at the aviary, whose VMR is indicated by the right y axes.

Many monoterpenes exist in two chiral forms, meaning they exist in nature as two non-superimposable mirror image forms (enantiomers), often with different biological activities. Insects can perceive each enantiomer differently and each may act as entirely distinct chemical signals^37^. We investigated the abundance of the prevalent chiral monoterpenes at the home aviary and at three ecosystems surrounding the aviary North (lake), East (mixed forest) and South (pine forest). This was to test whether unique mixtures could be ascertained at each site which is a condition of the mosaic hypothesis. At all sites, including the aviary, the (−) configuration dominated over the (+) for the monoterpene species α-pinene, β-pinene and limonene. This has been also observed in the tropical rainforest for α-pinene^38^. Relative ratios for the same monoterpene enantiomeric pair do not change significantly across the investigated sites, however, their absolute concentrations and relative abundance to the other terpene species do differ (see Fig. 3).

At the aviary, (−)limonene was the most abundant species (0.89 ± 1.7 ppbv and 2.5 ± 2.7 ppbv), followed by (−)α-pinene (0.42 ± 0.57 ppbv and 1 ± 1 ppbv), (+)limonene (0.26 ± 0.29 ppbv and 0.37 ± 0.42 ppbv) and (−)β-pinene (0.17 ± 0.45 ppbv and 0.27 ± 0.49 ppbv, values indicate mean daytime and nighttime values ± 1σ standard deviation, respectively). The lake site emitted the least monoterpenes, followed by the pine forest and the mixed forest, the latter showing the highest concentration of measured terpenes. Since (−)limonene dominates the terpene blend measured at the aviary, we can surmise that the surrounding pine forest had a strong impact on the air chemical composition at the site (Fig. 3). A similar suite of biogenic molecules was found at all locations, albeit with differing ratios. In other words, there were no clear unique chemical markers for a particular area. Therefore, the source of biogenic molecules near to the aviary has the potential to interfere with or mask, odours from more distant sites. These findings are not consistent with the original “mosaic” hypothesis in which each location in the region has a unique chemical signature that can be determined from the aviary.

### Olfactory maps: spatial and temporal distribution of VOCs

**Figure 4:**
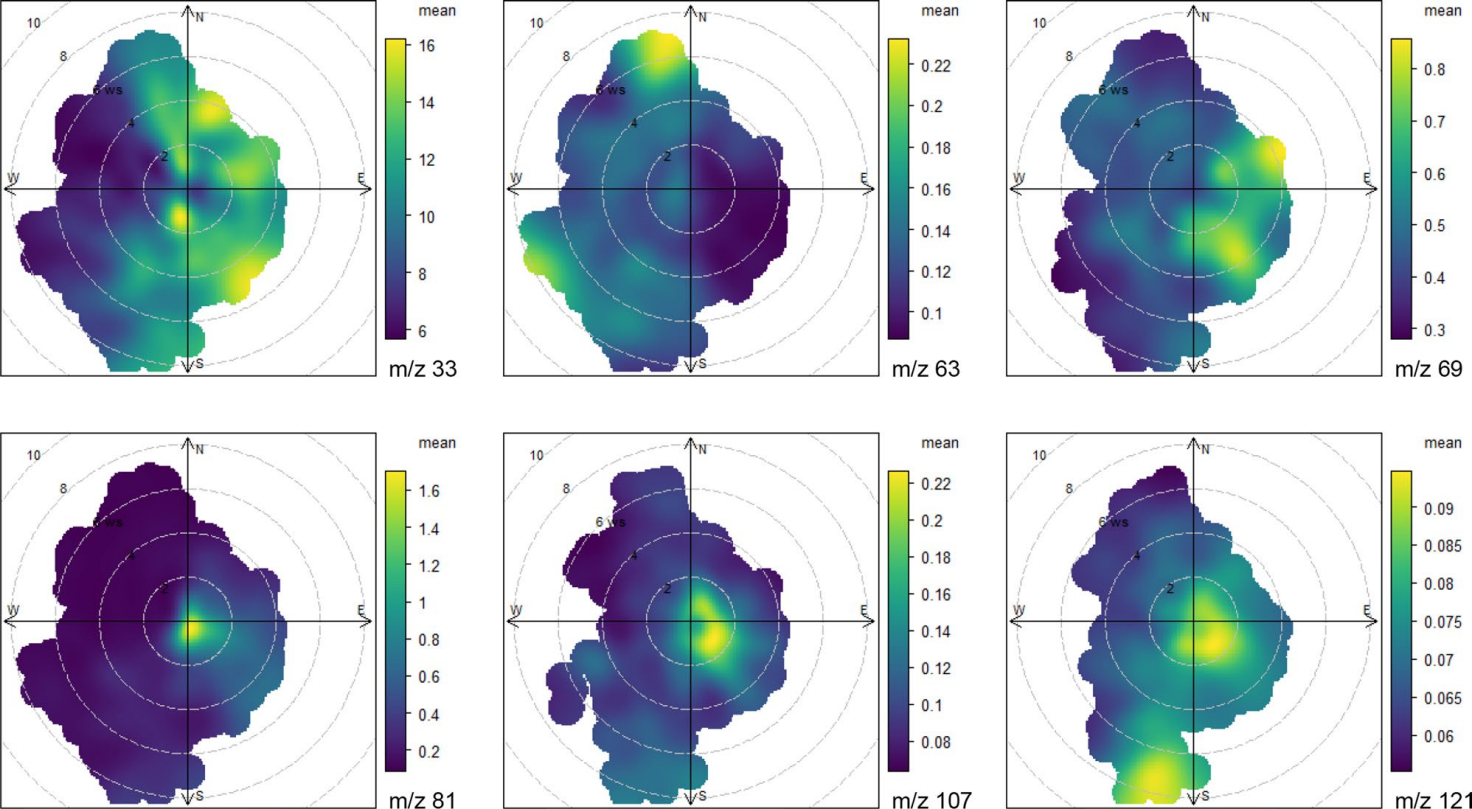
Spatial distributions of measured VOCs reported as protonated masses and identified as: methanol (*m/z 33*), DMS (*m/z 63*), isoprene (*m/z 69*), sum of monoterpenes (*m/z 81*), sum of xylenes (*m/z 107*), trimethylbenzene (*m/z 121*). Mean campaign values of VOCs (colored scale) are showed as a function of wind direction (angle) and wind speed (radius). Figures drawn with R.

Soon after fledging, the young pigeons are housed in a loft with an associated aviary, and from the end of May are allowed to perform spontaneous flights around the loft typically until October, when the season of the experiments usually ends. During the first months after fledging^39^ young pigeons are exposed to the changing chemical conditions at the home site and according to the Papi olfactory hypothesis, they learn to associate wind-borne odours with wind directions thereby generating an olfactory map. It is thought that this olfactory map is continually updated throughout their life depending on conditions experienced^40,41^. A visual representation of this spatio-temporal information can be expressed in the form of a bivariate plot of the measured volatile organic compounds. Bivariate polar plots represent the mean campaign mixing ratio of a given compound as a function of the wind direction (angle) and wind speed (radius), similarly to a wind rose they can highlight spatial gradients in the surrounding of a measurement site. Figure 4 shows that methanol (a biogenic compound and biomass burning marker) mixing ratios were higher for higher wind speed and easterly winds (from inland). However, mixing ratios were elevated at both high and low wind speeds; indicating some methanol sources were also local. Dimethyl sulphide mixing ratios were larger for higher wind speeds originating from the North–West and South–West sectors, spanning from the mouth of the river Arno to the sea. The largest mixing ratios for isoprene came from inland, in particularly from the West–North–East for higher wind speeds and the South–East for lower ones. Smaller mixing ratios are found from the sea for large wind speed (North–West), suggestive of a weaker marine isoprene emission source. Small marine isoprene emissions have been measured previously, especially in chlorophyll rich waters^12,33,42^. Monoterpene mixing ratios are higher at lower wind speeds, mostly for winds coming from South–East but also East–North–East and South–West. The polar graph highlights the multiple sources of monoterpenes, and that the levels at the site are also locally influenced. The anthropogenic aromatic species xylenes and trimethylbenzene were larger when the air was transported from inland, in particular from the South-East, where heavy-traffic roads such as highway E80, the road FI-PI-LI and via AureliaSS1 are located. Additional sources of aromatics appear South of the aviary for high wind conditions, especially for trimethylbenzene. Trimethylbenzene (C_6_H_3_(CH_3_)_3_), is an aromatic hydrocarbon characterized by a strong odour, which is generally isolated from the C9 fraction of aromatics during petroleum distillation. South of the aviary, in the Stagno industrial area, there is a large petroleum refinery (Fig. 1).

This air composition measured at the aviary is the air breathed and smelled by the birds during their first months after fledging. The birds are therefore likely to be aware of, mixed biogenic and anthropogenic sources to the South-East, a more biogenic dominated source (with differing composition) to the West-North–East, and a marine source to the West. Thus, the ground based measurement campaign mapped several chemical compounds that vary distinctly at the site according to the wind direction and time of day. Which VOCs are useful for navigation will also depend on their respective atmospheric lifetimes. Following emission to the atmosphere VOCs are oxidized mainly by OH radicals, with small contributions by O_3_, NO_3_ and Cl radicals. Methanol, the most abundant species measured, has a lifetime of 12 days (based on OH reactivity only, assuming OH radical concentration of 2 × 10^6^ molecules cm^–3^^43^). The corresponding lifetime of isoprene is 1.4 h, therefore with a typical windspeed of 2.2 m/s the concentration of isoprene will be reduced to 1/e of the initial value in 11 km, while limonene in 2 km, xylenes in 33 km and DMS in 88 km. Therefore, over distances of ca. 50–100 km, VOCs with moderate lifetimes such as DMS and the aromatic species appear capable of creating regional gradients. The more reactive VOCs are usually photochemically transformed into less reactive products which themselves, or their combination, may create regional gradients. Significantly, the three regional gradients highlighted in Fig. 4, namely DMS, the aromatics and the monoterpenes are not aligned, rather they slope in different directions. Theoretically then, by comparing the level of these compounds to that experienced at the aviary the pigeon may, with reference to the wind-odour experience at the aviary, orient homeward. For example if the DMS level is lower at the release site than at the aviary, and the bird “knows” from the olfactory map learning phase that DMS comes from the West, then the birds homeward direction will have a westerly component. Having multiple gradients available would enable the pigeon to triangulate a homeward direction.

### Regional spatial gradients

**Figure 5:**
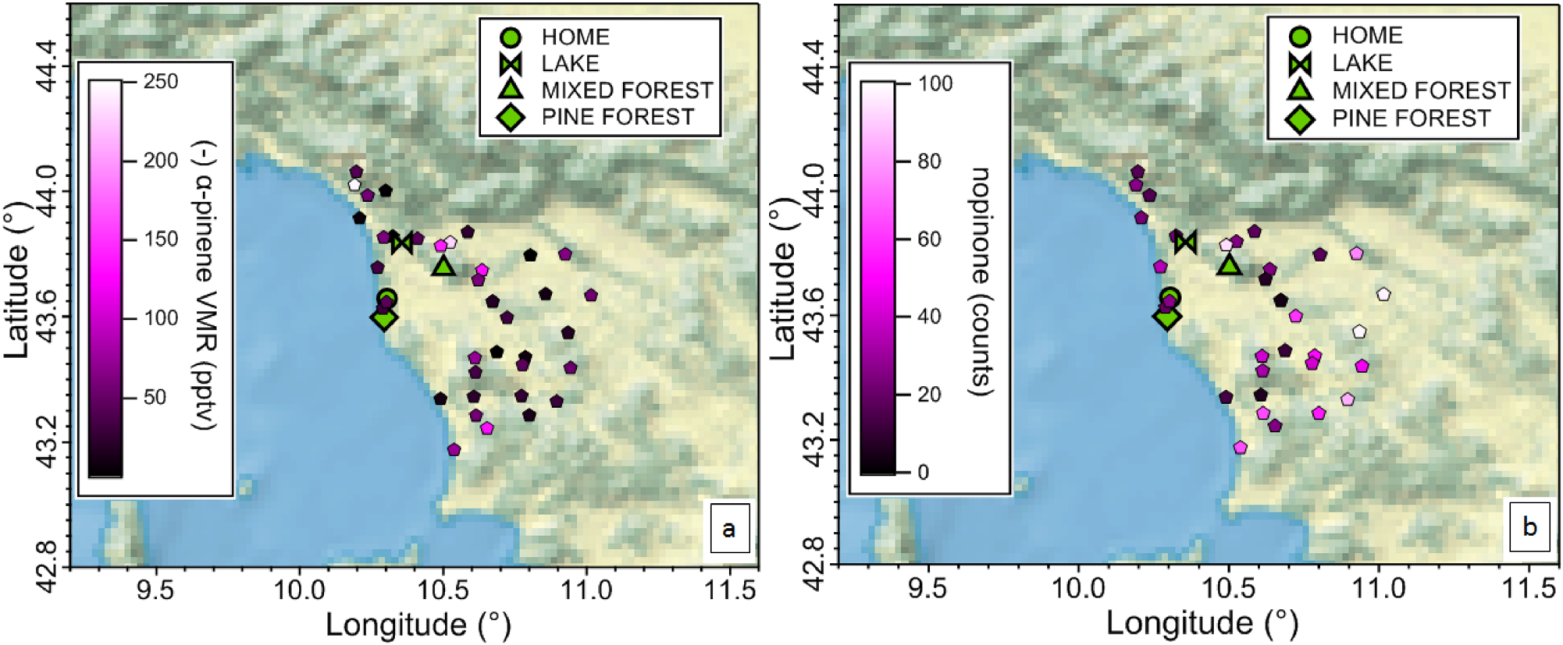
Spatial distributions of measured (−)α-pinene (**a**) and nopinone (**b**) from airborne sampling during three flights conducted on 26/05/2018 and 27/05/2018. Colored scales indicate respectively volume mixing ratio and counts of chromatograms peak area. Figures drawn with Igor WaveMetrics.

To verify that the spatial gradients observed from the olfactory maps exist at a larger regional scale we measured the air composition at the altitudes and over distances typically flown by homing pigeons (10–300 m, 100 km). Results from a flight campaign conducted on 26/05/2018 and 27/05/2018, in conjunction with the ground based campaign, are depicted in Figs. 3, 5 and Supplementary Fig. 3. Figure 3 shows the speciation of chiral molecules from ground measurements at four distinct sites (including the aviary) and above the sampled region considering all the airborne samples taken with the three flights. Interestingly, the regionally predominant compound at 180 m is α-pinene, rather than limonene observed at the ground. This is because α-pinene has a longer atmospheric lifetime (2.6 h), in comparison to β-pinene (1.8 h) and limonene (49 min)^43^. Figure 5 shows the mixing ratio of a biogenic precursor compound (−)α-pinene and the common terpene photochemical oxidation product nopinone^44^ sampled at different locations above the home aviary, and the release sites. The terpene airborne concentrations compare well with the ground-based ones (higher above the mixed Mediterranean forest, followed by the pine forest, followed by the lake site, as reported in Fig. 3). However, as expected, the terpene airborne concentrations are smaller compared to ground concentrations (40–50 pptv and 80–208 pptv, respectively) consistent with the ground based emissions being progressively oxidized by OH radicals and ozone as well as being mixed with the relatively clean air in the free troposphere above. This means that the birds will encounter strong vertical gradients of primary emitted species during flight with lower concentrations aloft. Furthermore oxidation chemistry will generate entirely different species, complicating the mixture, weakening the primary chemical signal and possibly obfuscating the originally emitted olfactory signals. In Fig. 5 we observe during the afternoon flights (with onshore winds) the nopinone mixing ratios generally increase to the East. This demonstrates that monoterpenes emitted at ground level during the day and in onshore winds are being oxidized to (among other species) nopinone on the time and space scales of the pigeon release experiments. Therefore, spatial gradients of reactive VOC do exist for the primary emitted species and their oxidation products, at a spatial scale compatible with the distances used for navigation experiments.

### Pigeon flight tracks and air masses trajectories analysis

**Figure 6:**
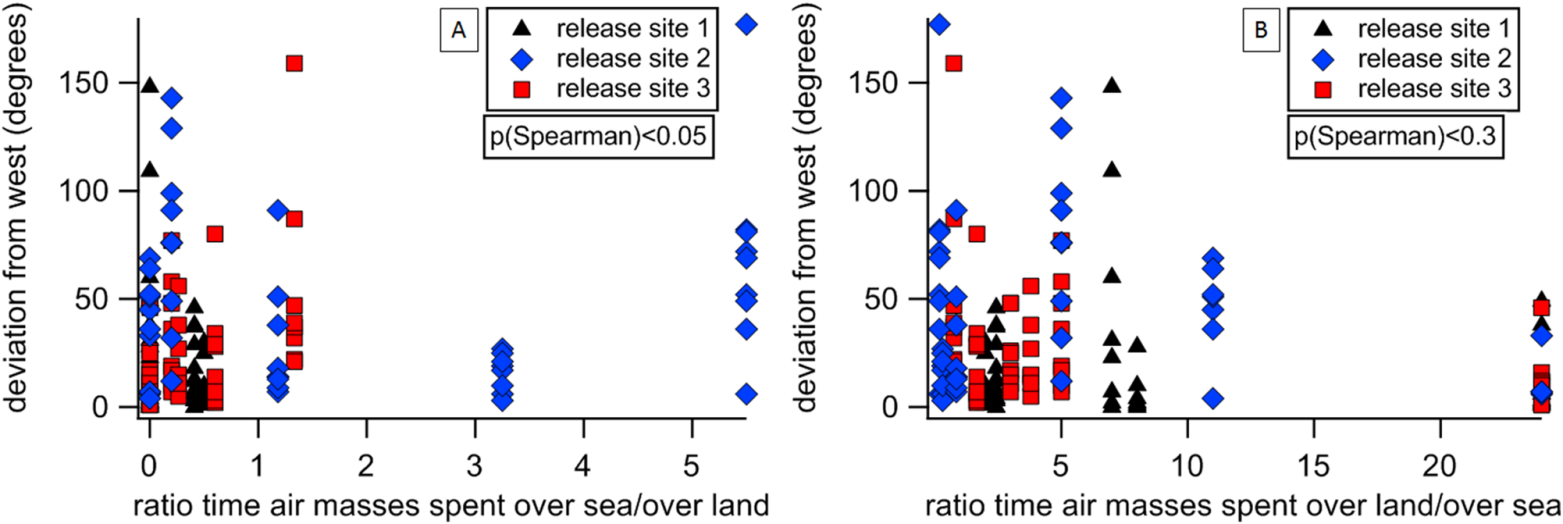
Deviation from west of initial orientation measured from flight tracks of individual birds released at the three release and plotted against the ratio the air masses at the release site spent in the marine boundary layer (above the sea) versus over land (**A**) and the opposite (**B**). This ratio indicates how much DMS is predicted to be in the air on a given occasion.

To test whether the intensity of atmospheric odour signals could potentially aid in the homing of pigeons, we provide here a first preliminary analysis based on real pigeon tracks and simulated air masses trajectories. We hypothesize that overall stronger gradients of indicative aerial chemicals enable better navigational performance in pigeons^18^. To do this we examine the GPS logged tracks of homing birds in relation to the general gradients of atmospheric chemicals determined in the previous sections, and to the origin of air at the release sites. Bird release experiments were conducted over six days in summer 2016 and summer 2017 from three release sites simultaneously (Fig. 1). Those experiments were chosen for being the most recent results available for indices analysis, and for being conducted on sunny days, when air temperature, humidity and atmospheric pressure were comparable to those encountered during VOCs measurements (Supplementary Fig. 4). Each release experiment involved 9 or 10 individual birds, released singly every 5–10 min, over approximately 1 h and involved 27–30 individuals released on the same day from three sites (N = 174). The total number of tracks obtained was 143, due to either the loss of the device by some birds or the misfunctioning of the GSM GPS logger. Bird flight tracks were used to determine: the pigeon initial flight direction, the homing capabilities en route with the homing efficiency index (HEI^45^) and for the whole track with a mean aggregate azimuth penalty (MAAP); see Methods and supplementary information. Due to lack of power and shelter at the release sites, chemical information of atmospheric composition was not available during those experiments, therefore a meteorological approach, based on modelled air trajectories, was developed in order to examine how air mass transport was related to the birds’ homing performance. Specifically, we generated forward and backward trajectories of the air masses for each release day, for the site and time of release. Forward trajectories analysis showed that the prevalent wind direction during the days and time of pigeons’ flights was from west (supplementary information). We cannot yet test the bouquet of potential chemical information, but concentrate here on one traceable chemical gradient: DMS (Dimethyl sulphide) emanating from the Tyrrhenian sea. DMS, a chemical compound of marine origin (see Supplementary Fig. 5), is here identified to be a suitable candidate for homing by olfaction. Figures 2 and 4 show that it was among the most abundant compounds measured during daytime at the aviary, it follows a regular pattern of emission, is known to be detectable by birds, and it is atmospherically stable enough to survive transport over longer distances, likely decreasing along the West–East direction. Air masses reaching the aviary diurnally during the ground campaign were found to spend considerable time (> 20 h in most cases) in the marine boundary layer in the past 24 h (see table 1 supplementary material). For these reasons, we tested if more marine-influenced air masses can influence the pigeon initial orientation (Fig. 6) and the homing path en route (homing efficiency index, supplementary material). We examined where each air mass was located (during 24 h prior to the release) reported in latitude and longitude coordinates and determined the time the air masses spent in the marine boundary layer, and the time the air masses spent over non-marine areas. As shown in Fig. 4, pigeons at the home loft are exposed to DMS associated with westerly winds; easterly winds are unlikely to carry DMS. According to the olfactory navigation hypothesis^5^ it is expected that a low atmospheric level of DMS at the release site is likely to produce a bias towards west in the orientation of the birds, regardless of their ultimate home direction. To test this hypothesis, the deviation from west of the individual mean vectors computed on the initial part of each track (within 10 km from the release site) is tested against the ratio of the time the air masses spent over sea versus over land (ratio sea/land) and the opposite (ratio land/sea). The Spearman ranking test highlighted that the deviation from west is positively correlated to the sea/land ratio of the air masses trajectories (n = 135, S = 0.163, p < 0.03), and negatively correlated to the land/sea ratio of the air masses trajectories (n = 135, S = − 0.171, p < 0.3). This difference in directionality is consistent with a role of the DMS gradient decreasing along the West–East axis indicating (to the birds) a displacement towards east.

We considered also longer bird flight trajectories (50 min of flight at a speed > 10 km/h) and the whole home path to test the birds homing performances with the indices HEI and MAAP. Generally higher homing efficiency indices were obtained from releases experiencing air masses originating from West of the aviary (Fig. 9, Supplementary material). This is supported by the correlation of homing ability with westerly wind component (see MAAP analysis in “Method” section, Supplementary Figs. 7, 8, 9 and Table 2). Depending on the considered release site and its direction with respect to home, significant trends exist between the homing path and the time the air masses spent in the marine boundary layer or above non-marine areas (supplementary Fig. 10 and Table 3).

**Figure 7:**
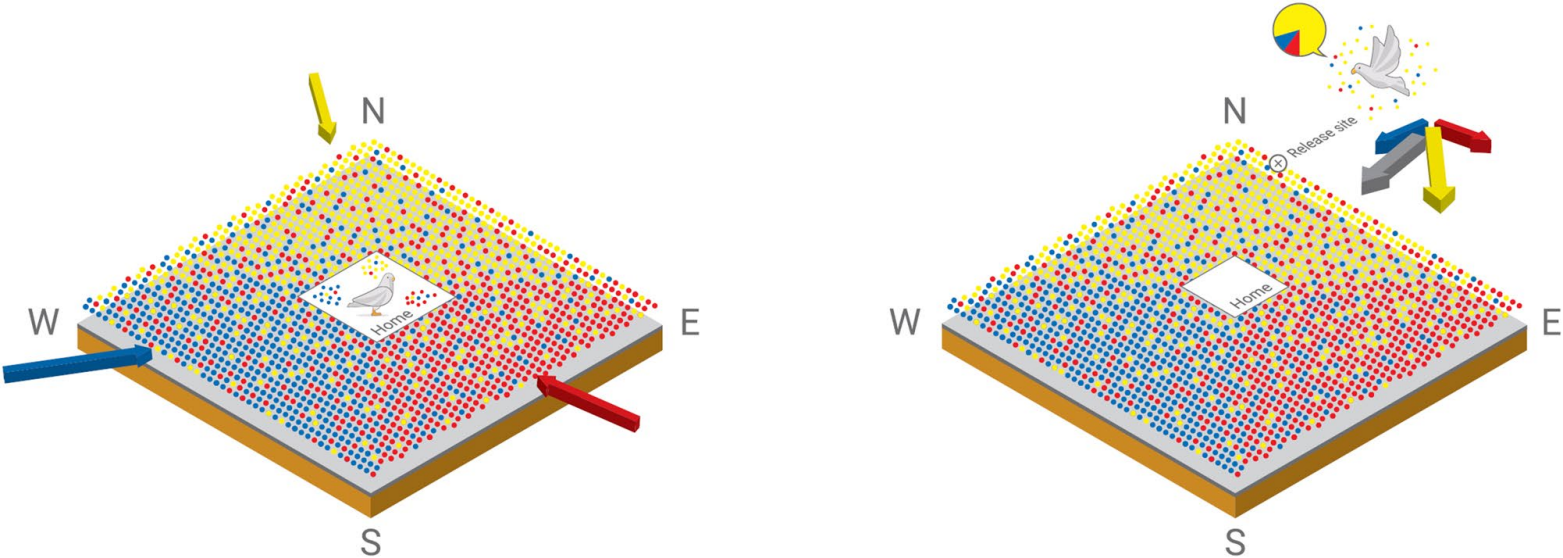
Over a period of months, fledgling pigeons learn to associate odours with wind directions and time of day. The coloured dots symbolize these regional scale odour gradients and the olfactory map envisaged by the pigeon (left panel). When pigeons are displaced to a release site (marked with a cross) the pigeon is exposed to the local odour spectrum (right panel). This compares to the odour spectrum experienced at home and orientates accordingly homeward. For instance a pigeon released in the yellow area will orient roughly south (yellow scent is higher at release point than at home and the pigeon knows this scent comes from the North). By triangulating a course from several regional odour gradients a homeward course can be determined (shown as the black arrow, the net result of the blue, red and yellow odours). Figure drawn with Adobe Illustrator.

### Potential biogenic candidates as olfactory cues

Previous studies on the role of olfaction in avian navigation focused mainly on alkanes and alkenes, compounds that are mainly of anthropogenic origin with atmospheric lifetimes ranging from days to weeks. Our study shows that biogenic compound distributions can also provide spatiotemporal varying chemical information suitable for bird navigation. Here, we draw attention to two classes of biogenic compounds with high navigational usage potential, specifically, DMS and terpenoids (isoprene and terpenes). DMS and terpenoids were both present at high levels at the bird’s aviary and due to the meteorology and chemistry strong regional gradients are established each day. In the present work, we observed that the main sources of DMS are located North and West of the aviary, while terpenes originated mainly from the East and South of the aviary and isoprene North–East–South the aviary. The concentration gradients of these species are therefore almost orthogonal to each other, potentially helping olfactory navigation.

Previous experiments have reported that the initial orientation and homing performances of pigeons from the same colony varied according to season, with the birds displaying better performances in spring/summer than autumn/winter^18^. This is consistent with the idea suggested here that biogenic compounds are involved in olfactory navigation since emissions of such compounds are driven by both light and temperature (as for terpenoids) and are therefore higher in summer than in winter. Dimethyl sulphide emissions also depend on photosynthesizing phytoplankton activity and seawater temperature; therefore, higher atmospheric concentrations are also seen during spring and summer, when the DMS concentration in seawater is also higher (see Supplementary Fig. 5).

## Discussion

Our study provides a comprehensive dataset of biogenic and anthropogenic volatile organic compound measurements from a rural site located in Italy, to investigate potential gas-phase navigation cues used by homing pigeons. For the first time, the air composition has been measured at a home aviary in conjunction with the development of fledgling pigeons, as well as in and above surrounding vegetation. Although the ground, air and forest sampling campaigns were conducted in separate years, the meteorology was sufficiently consistent to allow comparison. The long term data collected at the aviary shows the same range of temperature and humidity as observed in previous years and the day to day variability in wind direction and speed were small. Therefore each dataset can be taken as representative of the regional meteorological situation. Future work should include concerted measurements at the release site at the time of release as well as the aviary site. The VOCs measured at the aviary show a repetitive daily pattern associated with the regional sea breeze system, varying with wind direction, wind speed and chemical oxidation. These variations can form the basis of an olfactory map of the region that varies with time, and whose gradients can be exploited for navigation, confirming that the “olfactory map” based hypothesis is plausible. At the aviary, biogenic VOCs dominated, although anthropogenic compounds were also present and associated with certain directions (South). The measurements presented here show that regional chemical gradients exist West–East from DMS and North–South from terpenes and aromatics. The terpenes and aromatics provide orthogonally orientated regional gradients that could be used in conjunction with other regional gradients to navigate. Flight measurements confirmed that vertical gradients for terpenoids, and horizontal gradients for precursor pinene and oxidation product nopinone are observable at 180 m altitude. From the comparison of compounds measured in the three sampling site locations, we note that while relative mixing ratios change, many compounds are common. Thus, the original “mosaic” hypothesis, whereby remote sites emit site specific compounds can be rejected. This work shows clearly that emissions from any particular remote site when advected towards the aviary will be transformed photochemically and mixed in the vertical, as well as being combined with emissions from other ecosystems en route. This suggests that birds can use a combination of odours, based on what has been measured to be present in air in the region. Some chemicals may be preferred dependent on how well the pigeon smells the individual species.

A large body of evidence^39,45–48^ showed that local odours at the release site provide displaced pigeons sufficient map information, so to allow homeward orientation shortly after release. In fact, pigeons exposed to local environmental odours of the release site and made anosmic just prior release are able to orient homeward^39^. Along their way, pigeons might change their course and initial direction re-orienting on the basis of local visual landscape features^49–51^, and/or local odour perceived in the overflown area^45^. Fledgling birds fly only up to 800 m from the aviary. Therefore, although they become visually familiar with the local site they have no previous experience of the ca. 100 km distant release sites used. Previous experiments have shown that close to the aviary (4–6 km) pigeons revert to visual only navigation^52,53^. This study demonstrates that chemical emissions, oxidation and mixing all serve to establish regional gradients of chemicals detectable by pigeons. Exposure to these scents over months of repeating daily meteorological cycles allows the pigeons to associate odours with specific wind directions. Analysis of the birds’ flight tracks in combination with air transport patterns suggests that westerly air masses might affect initial orientation. Marine emissions, whose main marker is here represented by DMS, provide information of eastward displacement, offering a stable spatial regional gradient used to map their position relative to the aviary. This is represented schematically in Fig. 7. At the point of release, the pigeon must assess whether the local odour of a particular compound is stronger or weaker than that experienced at the aviary (left panel). If local levels are weaker, then the pigeon would orientate towards the wind direction it has learned is most strongly associated with this odour. If the release point has higher levels than at the aviary, then the pigeon will fly away from the direction associated with this odour, as is shown in Fig. 7 (right panel) for the odour symbolized by the yellow points. The initial orientation analysis reported in this work (Fig. 6) is consistent with the prediction that lower levels of DMS are associated with westward orientation (easterly winds are unlikely to bring high concentrations of DMS). However, a higher concentration of DMS at shorter distances from the coast might provide a clearer signal of a smaller eastward displacement and finally induce a more efficient movement towards home, northward or southward, depending on other VOCs gradient distributed along the North–South direction. This is consistent with the MAAP and HEI indices analysis (Suppl. material Fig. 8–10, Table 2, 3).

All the aforementioned chemical species have strong odours when perceived by human beings, however little is known about the perception of smells by birds^54^. Aromatic compounds are volatiles characterized by, even named for, their pungent odour while terpenes are often used in food and fragrance chemistry for their aroma. Dimethyl sulphide (DMS) is detected extremely sensitively by the human nose^55^ and its tangy odour has been used by sailors as a harbinger of land as DMS emission is strong in nutrient rich coastal waters. Seabirds are also known to use DMS as a foraging cue. Procellariform seabirds, including storm petrels, prions, petrels, and albatross are all sensitive to DMS and use it as an indicator of their main source of food^56^. There is also evidence that Humboldt penguins can use DMS to track upwind plankton blooms over long distances^57^.

Our data show that several orthogonally orientated chemical gradients exist that are compatible with olfactory navigation. Aviaries located elsewhere may be impacted by different VOCs, whose variability can affect the map learning process and homing ability. Thanks to the local sea breeze system at the Arnino aviary, each day the birds experience a repetitive pattern of mixed local vegetation and man-made emissions over wide wind angles and marine-influenced air with associated distinct chemical signatures. The fact that many species of birds are known to smell DMS, its atmospheric lifetime which allows regional transport and the establishment of gradients, and the observed trends which point to a more confident journey back home taken by birds upon release, make this molecule one promising potential olfactory navigation cue in this region. The combination with other gradient forming species permits a homeward course to be triangulated. Through this work a clearer picture has emerged of the specific chemical information exploited by the pigeon when navigating. Additionally, this study reports for the first time a clear analysis of the regional chemical composition and variability during spring–summer for a site where VOC information was before absent. The study also proposes a novel way of examining bird flight tracks in combination with meteorology, even though at this point we could only highlight one of several potential gradients in atmospheric chemicals that could be used in combination by the pigeons. Further studies should test the ability of pigeons to smell and recognize the compounds suggested here as candidate species. It should be recalled that the chemical information presented in this work came from several tons of ultrasensitive scientific instruments, making a ca. 400 g pigeons ability to decipher a complex local odour and interpret it in terms of regional gradients both spatially and temporally truly remarkable.

## Methods

### Experimental design of VOC measurements and site description

Volatile organic compounds (VOCs) were sampled in ambient air during three different studies: (i) a pilot study in which air samples were collected at three forested locations surrounding the measuring site, (ii) a 2 month long ground-based measurement campaign at the birds’ aviary in spring-early summer 2018, (iii) an airborne campaign consisting of three flights that ran simultaneously with the ground-based campaign.

For the pilot study, 2 L volume air samples were collected on adsorbent filled sampling tubes, five times a day, between 15/06/2017 and 19/06/2017 at three different sites from the same collection point. Technical details on the sampling are presented in the next section. The three sites were selected for being: (i) biogenic VOC dominated sites, located North, East, and South of the birds home aviary, (ii) characterized by different plant species that are widespread in the region. Specifically, the first site, to the North of the aviary, was Massaciuccoli lake (coordinates 43°50′10.9″N 10°21′23.2″E), a lake ecosystem within San Rossore and Massaciuccoli Natural Park. The second site, to the East, was the mixed Mediterranean forest located on Monti Pisani (43°45′15.3″N 10°30′06.5″E); and the third site to the South was the pine forest near Calambrone (43°35′49″N 10°17′40″E) (see Fig. 1). The dominant plant species at the lake ecosystem is the giant reed (*Arundo donax*), while the mixed forest comprises oaks (*Quercus suber, Quercus ilex*), chestnuts (*Castanea sativa*) shrubs and flowered species (*Erica sp., Cistus sp., Iris sp.* among the others). The pine forest is mainly composed of two types of pines: domestic pine (*Pinus pinea*), and maritime pine (*Pinus pinaster*). Sampling collection occurred at chosen collection spots far from specific sources (one tree or flower) and external sources (cars and humans). Samples were clear contamination occurred (a car passing by) were discarded and repeated before analysis.

The 2-month measurement campaign took place between May and June 2018, at the field station Arnino (Department of Biology, University of Pisa) hosting several home lofts with homing pigeons (*Columba livia*) (43°39′25.7″N 10°18′14.7″E). This site is rural, situated 1.8 km from the Tyrrhenian Sea on the western side, 3.2 km from the mouth of the river Arno to the North–West, about 11 km from the cities of Pisa and Livorno located on the northeastern side and southern side, respectively. Pisa (90,500 inhabitants) and Livorno (158,900 inhabitants) are the two main cities near the measurement site. The harbor of Livorno and the industrial area of Stagno (7.2 km south) are the main industrial areas nearby the site (see Fig. 1). The campaign consisted of comprehensive characterization of ambient VOCs using on-line Proton Transfer Reaction-Mass Spectrometry (PTR-MS) and Gas-Chromatography Mass Spectrometry (GC–MS). The instruments were located in an air-conditioned container installed on a site next to the aviary where the newly hatched birds were housed.

The airborne campaign consisted of in-flight sampling onto adsorbent filled sample tubes during three flights performed with the Max Planck Institute of Animal Behavior Cessna 172 aircraft on 26/05/2018 and on 27/05/2018. Flights left the airport of Lucca-Tassignano (45 km Northeast from Arnino) following circular tracks with different radii from the birds’ aviary, above the main release sites used when testing the birds homing abilities. Flights were conducted at 70 knots air speed at an altitude of approximately 500 feet (180 m) above ground, which is within the altitude typically flown by homing birds. Two-liter volume air samples were collected on adsorbent filled tubes as with the pilot study. A custom-built automatic sampler controlled the sampling flow.

### Field and laboratory experiments

#### Off-line sampling

Volatile organic compounds samples were collected by drawing ambient air through fused-silica-lined stainless steel 3.5″ (89 mm × 5.33 mm I.D.) sampling tubes containing two adsorbent beds [180 mg Tenax TA followed by 130 mg of Carbograph 1 provided by Alltech (USA) and Lara s.r.l. (Italy)] as described in detail by Kesselmeier et al.^58^. The sampling flow was held constant at either 200 mL/min or 250 mL/min and the sampling time varied according to the tube resistance, in order to collect 2 L volume samples. A filter impregnated with a solution of 10% w/w sodium thiosulfate prepared in the laboratory was used to scrub ozone from the sampling flow. The sampled tubes were stored at cool temperature (5 °C) and analyzed within 1 month. The analysis was performed in the laboratory with a Thermodesorption Gas Chromatograph system equipped with a Time of Flight Mass Spectrometer (TD-GC-TOF-MS, Bench ToF Tandem Ionization from Markes International, UK). The TD-GC-TOF-MS first desorbs the analytes from the sampling tubes in two sequential stages, both performed at 250 °C for 10 min. The desorbed analytes are swept in a flow of He into the separating column housed in the gas chromatograph. The column was a dimethyl TBS β-cyclodextrin based column (0.15 µm, 0.15 mm ID, 25 mL, from MEGA, Italy) which separates the analytes according to volatility and enantiomeric configuration. The separation method was specifically designed for the separation of chiral monoterpenes (C_10_H_16_) and consists of an initial 5 min when the oven temperature was held at 40 °C, after which it was increased at a rate of 1.5 °C/min from 40 to 150 °C. Finally, the temperature was increased further at a rate of 30 °C/min from 150 to 200 °C. Detection was performed by a Time of Flight Mass Spectrometer, which fragments the analytes through electron impact ionization at − 70 eV for quantification and identification of the chemical species. Identification of the main chemical compounds was obtained by comparing the MS spectra with the MS library for the same ionization energy (NIST library), by injection of a certified gas standard mixture (162 VOCs provided by Apel-Riemer Environmental Inc., USA) and by use of certified liquid standards. Chromatogram peak areas were integrated through the TOF-DS software provided with the instrument.

#### On-line sampling

A 1.27 cm (½ inch) OD Teflon insulated and heated main sampling line was installed on top of an air-conditioned laboratory container. A sampling pump continuously pulled 10 L/min flow of air to the two instruments (gas chromatography–mass spectrometry and proton transfer reaction- mass spectrometry) placed inside the container. The sampling inlet was equipped with a particle filter, a downward facing funnel to protect against rain, and a net to prevent insects being pulled inside the sampling lines. An extra particle filter previously treated with a solution 10% w/w of sodium thiosulfate was placed before the gas chromatograph in order to scrub ozone from the sampling flow.

### Thermodesorption gas chromatographer mass spectrometer (TT-GC-MS)

The TT-GC-MS consisted of two units; a thermodesorption unit (TT24-7 from Markes International, UK) equipped with two sampling traps that operates by alternately sampling and desorbing the VOCs in ambient air, and a GC–MS (G6890A, from Agilent Technologies, USA). A Kori-xr (from Markes International, UK) system was used to remove water from the sampling flow prior to analyte focusing. The sampling flow on the two traps was held constant at 200 mL/min for 15 min; hence, 3 L was collected on each trap and subsequently desorbed in a flow of He which carried the analytes into the gas chromatograph. The on-line GC was equipped with the same chiral-based column as the TD-GC-MS used for the offline sampling, separating enantiomers according to their boiling point with the same method. Finally, the chemical compounds were analyzed through a quadrupole mass spectrometer (Agilent Technologies), operated with − 70 eV electron impact ionization. Identification and quantification of the VOCs was achieved through MS spectra comparison with the NIST library and through injection of a certified standard gas mixture (162 VOCs from Apel-Riemer Environmental Inc., USA). Chromatogram peak areas were integrated through the software Mass Hunter provided by Agilent Technologies.

### Proton transfer reaction-mass spectrometer (PTR-MS)

The proton transfer reaction-quadrupole mass spectrometer (PTR-QMS, Ionicon Analytik, Austria) was deployed in the campaign to monitor VOCs with a high time resolution (5 s dwell time for each selected mass fragment) together with the meteorological values. The principle of PTR-MS is described in detail in Lindinger et al.^59^. We operated the PTR-MS with standard settings (H_3_O^+^ as primary reagent ion, drift voltage 600 V, drift pressure 2.2 mbar and drift temperature 60 °C), high primary ions H_3_O^+^ signal (2.4 × 10^7^ cps), low first water cluster signal (< 4% of H_3_O^+^), low NO^+^ (< 3% of H_3_O^+^) and low O_2_^+^ (< 4% of H_3_O^+^). Supplementary Table 4 reports the selected mass fragments, their attributed identity and limit of detections for this study. Instrument calibrations were performed under different humidity levels (tracked as *m/z *37/*m/z *19), with a certified gas standard mixture provided by Apel-Riemer Environmental Inc., USA containing the chemicals listed in Supplementary Table 4. Background measurements were conducted regularly using synthetic air (Westfalen AG, Münster, Germany) for about 1–1.5 h and the actual ambient mixing ratios were obtained by subtracting the measured background values. As PTR-QMS does not measure compounds at their exact mass, we ruled out possible interferences on the measured fragments by comparison with online GC–MS and available literature. *m/z *69, common fragment attributed to isoprene, was corroborated by isoprene data acquired simultaneously by GC–MS with a good least-square fit (slope 0.80, R^2^ = 0.85). Jardine et al. (2015) pointed out the interference of protonated acetaldehyde water cluster (CH_3_CHOH^+^-H_2_O) on *m/z *63, usually attributed to dimethyl sulfide (DMS), was further demonstrated experimentally to be negligible. Considering that (i) the same operational settings of PTR-MS were used as Jardine et al.^24^ and that (ii) both acetaldehyde and DMS were available in the gas mixture and quantified with repeated on-field calibrations under different humidity levels, we attributed *m/z *63 solely to DMS.

### Meteorological data

Weather parameters were collected every second from a portable weather station (WXT530, Vaisala, Finland) installed on top of the container. The logged parameters included temperature, humidity, wind speed, wind direction and rainfall.

### Bivariate polar plots

Wind speed, wind direction and mixing ratio data are partitioned into wind speed direction bins and the mean mixing ratio calculated for each bin, following Carlslaw and Ropkins^60^.

### Air mass trajectories and related analysis

Backward and forward trajectories of air masses reaching the release sites were modelled using the online version of HYSPLIT (HYbrid Single-Particle Lagrangian Integrated Trajectory) developed by the National Oceanic and Atmosphere Administration (NOAA) Air Resources Laboratory, see Stein et al.^61^ for more information. Trajectories were modeled forward and backward for 24 h from the time of birds release at the release sites coordinates. An altitude resembling the birds flying altitude (150 m) was chosen as ending point. Mixing layer depth data were extracted at each hour for each modeled trajectory. Trajectories data were filtered according to their transport altitude and coordinates in: air masses transported over the sea, over the land and above the boundary layer height.

NOAA archived weather data were used to generate the average wind profiles for 24 h from the bird release sites, as most of the bird data is also available for up to 24 h (i.e., either the birds reached the aviary within 24 h or the birds were lost within this time window). The wind profiles provide three pieces of information: the mean wind direction, the mean wind speed, the percentage of time a specific strength wind was coming from a given geographical direction.

Using this information, we defined a west wind component (WWC) feature such that west winds get the value + 1, east winds get the value − 1, the western hemisphere gets positive values for the west wind component and the eastern hemisphere gets negative values.

The geographical direction was encoded as sixteen equi-distant values between 0 and 2π denoted by θ, such that 0 is aligned with west and π is aligned with east. Next, we took the cosine of the angle values that gives us a column vector.

Based on the NOAA wind profile for each release, we extracted the ratio of time the wind was coming from a particular geographical direction and also the average wind speed to get a matrix of 16 × 6 such that each entry in the matrix corresponds to a specific geographic direction and a specific wind speed.

We used this information to calculate the West wind component for a specific date and location by summing the product between the cosine of angles, the ordinal wind speeds’ vector (S) and the wind profile matrix (P):

$$ WWC = \sum {(cos{\theta  } \times {\text{ S }} \times {\text{ P}})} $$

This quantity is equal to the expected value of the strength of the West wind component.

### Bird release and homing indices

One hundred and seventy-four unexperienced homing pigeons (*Columba livia*) of both sexes, hatched in the same year of the test at the Arnino field station (latitude, 43°39′26″N; longitude, 10°18′14″E), Pisa, Italy, were used in the study. The pigeons were raised as free flyers and were allowed to fly around the loft but never displaced before the experiments. It was observed that they do not fly further than 800 m from the loft^53^, so the release sites were unfamiliar to them. Before each displacement experiment homing pigeons were tagged with GPS GSM loggers (Quectel M66) transmitting to a platform (Telepath) sampling 1 fix every 10 s, recording latitude, longitude, flight speed and time. Birds were released from unfamiliar sites at different compass directions but similar distance from home (see Fig. 1). Specifically, release site 1 is found to have a bearing of 241° to home and distance 59.9 km; release site 2 at 309° and 50.5 km while release site 3 at 270° and 61.0 km. We determined the initial (within 10 km radius from the release point) flight direction as individual mean vectors for each individual pigeon and relate this to the deviation from west. The mean vectors with r (mean vector length) lower than 0.1 were excluded from the analysis because such small vectors indicate that a particular bird did not show a consistent orientation. For each track from the release point up to the fix corresponding to 50 min of active flight (speed > 10 km/h), or to home if the bird reached home within this time, the Homing Efficiency Index (HEI), was also calculated^45^. The HEI expresses how accurately the birds headed homeward when actively flying. This parameter takes into account both the tortuosity of the flight path and whether a bird approaches or flies away from home during its flight. The HEI can be calculated as follows:

$$ HEI = \frac{a}{l} \times \frac{{\left( {b - c} \right)}}{b} $$

where a is the beeline between the release site and the last fix recorded, l is the track length, b is the beeline between the release site and home, and c is the beeline between the last fix recorded and home (in km). Therefore, a pigeon failing to approach home gets a negative HEI, while a bird approaching home gives a positive HEI. The median HEI of the group of birds released from each site on each day was considered during the time of active flight (113 individuals for the considered time). A second analysis was applied specifically to examine the dependence of the West wind component on homing ability. This included the distance from the aviary for each bird (distance between aviary and last recorded point in each flight) and the mean aggregate azimuth penalty (MAAP) value for each bird flight trajectory. The MAAP measure is calculated using the actual heading/azimuth of the pigeon and the desired heading/azimuth to reach the aviary, taken at regular intervals (one hour in the reported calculations) over the flight path so that no single point dominates the calculation of the MAAP measure. Next, for each sample in the bird flight, we calculated the desired azimuth ‘A’, required for a direct flight to the aviary and the observed azimuth ‘a’, which is the actual heading of the bird. The sum of the absolute differences between each corresponding pair of the required azimuth ‘A’ and the actual azimuth ‘a’ is calculated and normalized with the length of the down sampled bird flight data. Formally, a bird flight is reduced to only hourly samples and the sum of absolute differences of the desired azimuth ‘A’ and the observed azimuth ‘a’ for each hourly sample is calculated and normalized by the number of hours the flight took place. Length normalizing the MAAP value allows comparing the different flight observations. This is also useful because the in some hourly intervals, the pigeons may fly relatively far but not very far in another interval. For a trajectory mostly conforming to a straight line trajectory homeward, the penalty value is close to zero, while for a flight trajectory which was extremely off the straight line path the penalty value is high. Effectively, this quantity measures how much, on average, the pigeon deviates from the ideal flight route; as such any signal loss or small detour but further flight ending point are not penalized by our analysis.

### Ethics

Pigeons were kept and treated according to the Italian law on animal welfare. This research was authorized by the Italian Ministry of Health (permit number 1019/2015-PR).

## Supplementary information

Supplementary Information.

## Data Availability

The datasets generated and analyzed during the current study are available at https://github.com/kramerlab/olfactory-mapping and in the Movebank Data Repository, 10.5441/001/1.7n747m19.

## Abbreviations

Aromatic compounds: Hydrocarbons having an aromatic ring (cyclic structure with resonance double bonds) in their chemical structure. Benzene and derivatives (xylenes, toluene, trimethylbenzenes) are examples
DMS: Dimethylsulphide (C_2_H_6_S). Chemical compound of natural origin, whose main source in the atmosphere is from marine phytoplankton. It is characterized by a tangy smell
Enantiomers: Isomers whose structures are the non-superimposable mirror image of each other. They can also be called chiral, distinguishable only by how they rotate plane-polarized light. Example: (-)limonene and ( +)limonene.
GC–MS: Gas chromatography Mass Spectrometry, analytical instrument commonly used to detect specific chemical compounds in gaseous samples. The sampled mixture is first separated through chromatography into its chemical components, which are then ionized through electron ionization and detected in the mass spectrometer. It is regarded as a hard ionization method (typically 70 eV), and often considered the technique of choice when specificity and chemical resolving power are requied
HEI: Homing efficiency index. It expresses how accurately the birds head homeward when actively flying
Isomers: Molecules or ions with identical chemical formula but different structure. Example: the monoterpenes alpha pinene and limonene (C_10_H_16_) are isomers
m/z: mass-to-charge ratio. It is a physical quantity commonly used as a dimensionless unit in mass spectrometry to define the mass to charge carried by an ion or a molecular fragment of a chemical compound. Specifically, in a Proton Transfer Reaction Mass Spectrometer operating at standard conditions, the sampled molecules are ionized via proton transfer reaction by the primary ion H_3_O^+^ to molecular ions. By applying an external electrical field to the drift tube, molecular ions can travel to the detector and be quantified. Fragments with specific *m/z* represent the chemical fingerprint of a molecule. Identification of fragments with specific *m/z* is achieved by using certified standard gases and the existing literature
MAAP: Mean aggregate azimuth penalty. It is calculated using the actual heading/azimuth of the pigeon and the desired heading/azimuth to reach the aviary
MEK: Methyl ethyl ketone. (C_4_H_8_O) An oxygenated volatile organic compound containing a ketone moiety
MVK + MACR + ISOPOOH: (C_4_H_6_O) Methyl vinyl ketone, methacrolein and isoprene peroxides. Three products resulting from isoprene oxidation with the hydroxyl radical OH, all measured at the same mass in the PTR-MS
ppbv: parts per billion by volume of air (volume of gaseous compound per 10^9^ volumes of ambient air). It is a unit used to indicate the volume mixing ratio of a chemical compound in air. For example, 1 ppbv of benzene, at standard temperature and pressure corresponds to a concentration in air of 3.19 μg/m^3^
pptv: parts per trillion by volume of air (volume of gaseous compound per 10^12^ volumes of ambient air). As ppbv, pptv is also used to indicate atmospheric mixing ratios of trace gases in ambient air
PTR-MS: Proton Transfer Reaction Mass Spectrometer, analytical instrument commonly used for monitoring atmospheric gases at high frequency. The technique is based on the physical process of ionization of a gaseous compound via a proton transfer reaction. It is regarded as a soft ionization method, and often considered the technique of choice for its ability in detecting trace amounts of chemicals with a fast response
TD-GC-TOF–MS: Thermodesorption- gas chromatography- time of flight- mass spectrometry. The GC–MS is equipped with a thermodesorption (TD) unit prior to the gas chromatograph (GC) and a time of flight (ToF) detector in the mass spectromter (MS)
Terpenoids: Hydrocarbons belonging to the terpene class (monoterpenes, sesquiterpenes, diterpenes, etc.…). These molecules have the characteristic of being composed by building blocks of isoprene (C_5_H_8_), have natural origin and a strong smell. Monoterpenes (C_10_H_16_) include among the others, α-pinene, β-pinene and limonene
TT-GC–MS: Tandem thermodesorption- gas chromatography- mass spectrometry. The GC–MS is equipped with a unit which serves to desorb the analytes from the sample and operates in tandem, making the measurement continuous
VMR: Volume mixing ratio. It indicates the ratio between the number density of a substance in a given number density of air molecules
VOC: Volatile organic compound. A gaseous species found in the atmosphere in trace amounts (pptv-ppbv)
WWC: West wind component. Parameter used to determine the strength of the westerly wind in a forward trajectory of an air mass. It takes into account the wind direction and speed at a specific time and place
