# Supplementary material for: Identifying volatile organic compounds used for olfactory navigation by homing pigeons
Source: Sci Rep. 2020 Sep 28;10:15879. doi: 10.1038/s41598-020-72525-2 (PMC7523013; doi:10.1038/s41598-020-72525-2)
Supplement: Supplementary file 1 — Supplementary Information. [file 41598_2020_72525_MOESM1_ESM.pdf]

Supplementary information for:

"Identifying volatile organic compounds used for olfactory navigation by homing pigeons"

Nora Zannoni[1*], Martin Wikelski[2,3], Anna Gagliardo[4], Atif Raza[5], Stefan Kramer[5], Chiara Seghetti[1], Nijing Wang[1], Achim Edtbauer[1], and Jonathan Williams[1]

[1] Max Planck Institute for Chemistry, Department of Atmospheric Chemistry, Mainz, Germany

[2] Max Planck Institute of Animal Behavior, Department of Migration, Radolfzell, Germany

[3] Centre for the Advanced Study of Collective Behaviour, University of Konstanz, Konstanz, Germany

[4] Department of Biology, University of Pisa, Pisa, Italy

[5] Department of Computer Sciences, Johannes Gutenberg University, Mainz, Germany

*corresponding author: nora.zannoni@mpic.de

Regional observations of volatile organic compounds

The meteorological parameters monitored during two months of intensive field campaign in Arnino are reported in Fig. 1. Temperature and relative humidity (bottom plot, Fig. 1) had a repetitive and mirrored pattern for the whole campaign. Wind speed and wind direction (top plot, Fig. 1) had also a repetitive pattern due to the influence of the local sea-breeze system at the site.

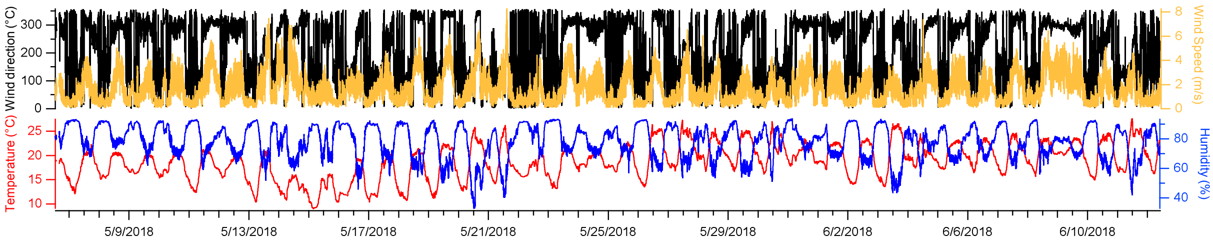

Figure 1. Meteorological conditions during the ground-based measurement campaign occurred in spring-summer 2018 at the field site Arnino (Italy).

Diel variations of volatile organic compounds (VOCs) are showed in Fig. 2 in the main text and Supplementary Fig. 2, below. Supplementary Fig. 2 show the diel profiles of the protonated mass fragments *m/z* 33, *m/z* 42, *m/z* 45, *m/z* 61, *m/z* 71, *m/z* 73, *m/z* 79, *m/z* 93, *m/z* 121, attributed respectively to methanol, acetonitrile, acetaldehyde, acetic acid, sum of methyl vinyl ketone (MVK) methacrolein (MACR) and isoprene peroxides (ISOPOOH), methyl ethyl ketone (MEK), benzene, toluene and trimethylbenzenes. Methanol, which is emitted from vegetation and biomass burning, had the highest concentration of all the measured compounds (12.2 ±6 ppbv, max mean value ± 1 σ standard deviation during the campaign), peaking at 10:00 and 21:00 (local time), with a diel profile showing maximum values when the air masses were coming from the South-East. A similar profile is observed for acetonitrile, whose emission is exclusively due to biomass burning. Acetonitrile mixing ratios were highest at 9:00 and 20:00. In contrast, isoprene (Fig.2 main text) maximum mixing ratios were at 11:00- 12:00, and 18:00- 20:00; similar to MVK+MACR, acetic

acid and MEK, which all showed one peak in the morning and one in the evening. Isoprene mixing ratios increase in the morning, as the plant emissions are triggered by increasing temperature and light, and the concentration of the main atmospheric oxidant (OH radicals) is low. With increasing incidence of light and temperature, although the isoprene emission rates from the vegetation increases, so does OH production and the atmospheric boundary layer height, both of which act to lower ambient isoprene mixing ratios measured at the ground. After noon, the isoprene emission rates begin to decrease, as do the mixing ratios of isoprene driven by continued oxidation photochemistry and turbulent vertical mixing within the boundary layer. Then unusually, isoprene mixing ratios increase again, peaking between 18:00- 21:00. It is important to recall that the measuring site is affected by the sea-breeze system described above; and that off shore winds (150°) prevail in the morning and later in the afternoon onshore (270°) between 12:00 and 20:00 dominate. Indeed, the second peak in the isoprene (evening) is correlated with DMS suggesting that it is related to this flow regime. Possibly marine influenced air from the west (containing DMS) has passed over a forested area during the strongest emission period before reaching the site. Alternatively, air from the land containing isoprene has been advected out over the sea in the morning, where DMS emissions mix in, then advected back onshore in the afternoon. Around 18:00 the boundary layer becomes shallower (Garratt, 1994), lowering the effective volume of air into which any residual emissions are emitted. Thus, even weak continued emissions from the forested areas to the West of the site can contribute to the second isoprene mixing ratio maximum. Similar reasoning holds for the oxygenated compounds: acetic acid, MVK+MACR and MEK.

Acetic acid and MVK+MACR are oxidation products of the isoprene reaction with OH, therefore a similar behaviour slightly shifted in time is expected. MEK can be emitted directly from mixed biogenic- anthropogenic sources (Yanez-Serrano et al., 2016), and this species shows different behaviour. The MEK mixing ratio increased at 7:00, again at 11:00 and again at 18:00, which

suggests different sources of emissions compared to isoprene. Acetaldehyde can be directly emitted from biogenic sources but can also be formed via the oxidation of biogenic VOCs. Here, its mixing ratio remained approximately constant during the night and morning, decreasing after 12:00 to increase again after 20:00. Again, this is associated with local meteorology and the fetch over biogenic sources. Acetaldehyde levels are lowest when the wind comes from the sea and highest when from inland.

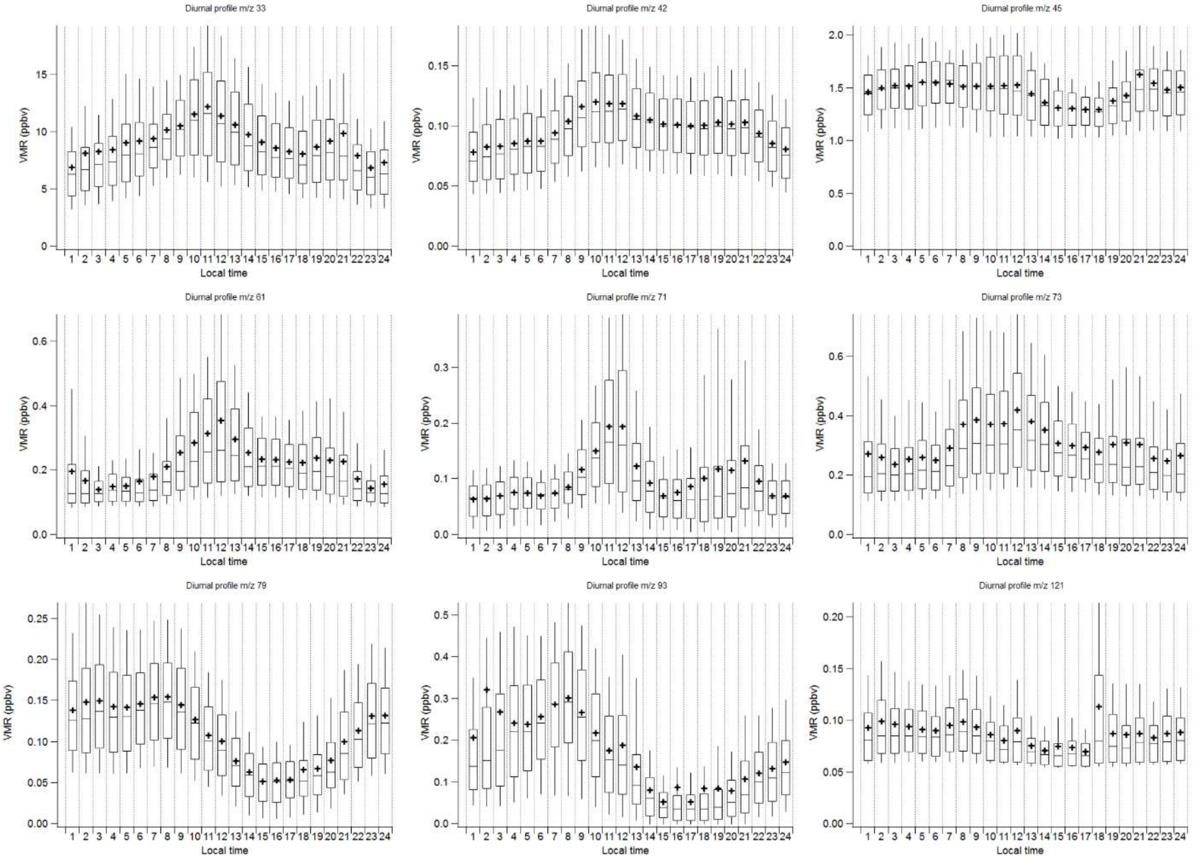

Figure 2. Diel profiles of *m/z* 33, *m/z* 42, *m/z* 45, *m/z* 61, *m/z* 71, *m/z* 73, *m/z* 79, *m/z* 93, *m/z* 121, attributed respectively to methanol, acetonitrile, acetaldehyde, acetic acid, sum of methyl vinyl ketone (MVK) methacrolein (MACR) and isoprene peroxides (ISOPOOH), methyl ethyl ketone (MEK), benzene, toluene and trimethylbenzene. Data were taken during two months of intensive field campaign at the field site Arnino (Italy) where the birds' aviary is placed. Box plots report mean campaign values (crosses), median (line), interquartile range (box) and 10th and 90th percentiles (whiskers).

From the airborne sampling conducted at 180 m above the region, a North-South gradient was observed for (-)α-pinene/(+)α-pinene; (-)limonene/(+)limonene and nopinone (counts). Figure 3 shows results from the sampling conducted on 27/05/2018 for (-)α-pinene/(+)α-pinene, (-)limonene/(+)limonene, and nopinone. The concentration increased when travelling from North to South.

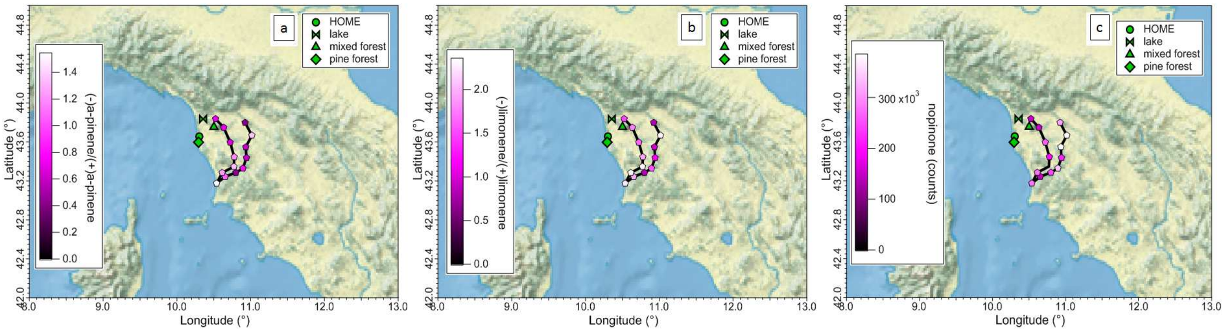

Figure 3. (-)α-pinene/(+)α-pinene (a), (-)limonene/(+)limonene (b), and nopinone (c) concentrations expressed as ratios of optical isomers and counts of peak area measured by gas chromatography. The sampling occurred during the second flight on 27/05/2018. Colored data points indicate each sample collected from the plane, the black line represents the flight track while the green markers indicate the birds' aviary (home) and the three sites of sampling investigated during the pilot study in summer 2017 (see methods). Figures were drawn with Igor WaveMetrics.

Bird flights performances

In order to test potential candidates for homing and bird flights performances we considered the most recent flight tracks collected from pigeons release experiments. Release experiments were conducted on sunny days during August 2016, July 2017, August 2017 and September 2017, when diel cycles and values of temperature, pressure, and relative humidity were similar to those measured during the ground and airborne campaigns (May-June 2018) and the pilot study (June 2017), see supplementary Figure 4.

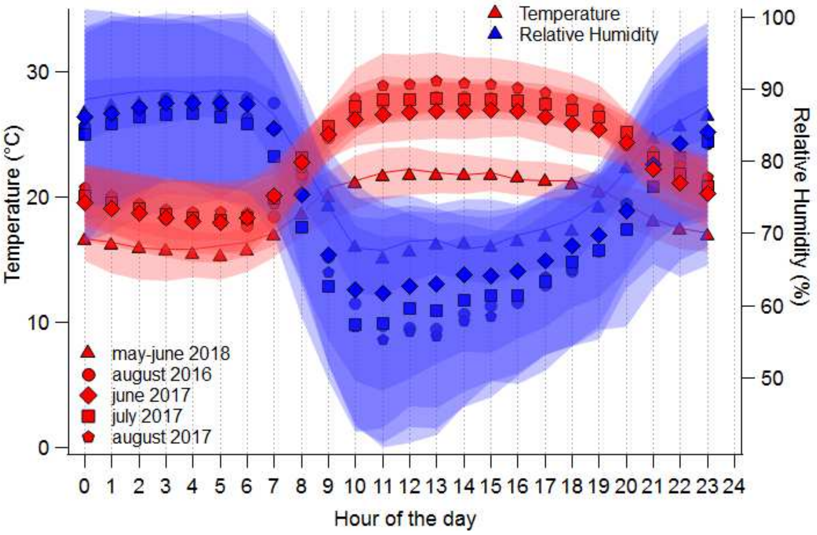

Figure 4. Temperature and relative humidity data collected from the permanent weather station installed at the aviary during the months when the birds release experiments, the pilot study, the ground and airborne campaign were conducted. Markers indicate mean monthly values while the shaded areas represent the respective 1σ. For the ground campaign T and RH data from the auxiliary weather station installed for the campaign are plotted. In this case, lines indicate median campaign values, markers mean campaign values and shaded areas the respective 1σ standard deviation. Data from September 2017 were not available.

We cannot yet test the bouquet of potential chemical information, but concentrate here on one traceable chemical gradient: DMS (Dimethyl sulphide) emanating from the Tyrrhenian sea. Dimethyl sulphide (DMS) atmospheric concentration directly depends on sea plankton activity and dynamics and air temperature and wind speed. Here, we modelled the DMS climatology according to the model provided by Lana et al., 2011 for the spring-summer months. Concentration of DMS in seawater peaked during May, June and August, associated to the blossoming season and higher ambient temperature. Therefore, higher concentration of atmospheric DMS is also expected during May, June and August.

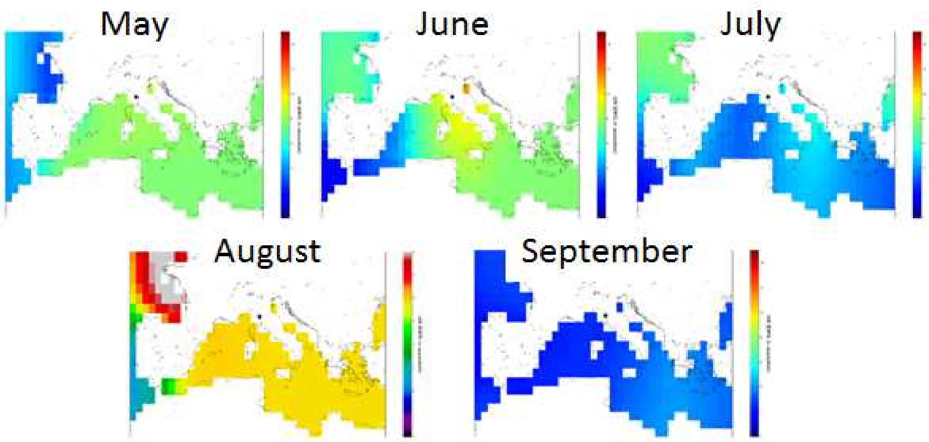

Figure 5. Modelled Dimethylsulfide (DMS) climatology across the spring-summer months. The colored scale indicates the concentration expressed in nM of DMS in seawater, while the star indicates the position of the birds'aviary.

We tested that fledged birds at the aviary experienced similar transported air masses as release birds during the considered experiments. We modeled 24h backward air trajectories reaching the aviary (coordinates 43°39'25.7"N 10°18'14.7"E) at 16:00 local time, at the ground where the instruments were located. For each inspected day, the output provides 24 data points of coordinates of air masses, altitude of the air masses and atmospheric boundary (mixing) layer height. Points above the boundary layer height were discarded. The other data points were filtered according to the coordinates of the air masses to check how long the air masses spent above the sea or above land. Table 1 shows that for all the inspected days of the campaign period, the air masses were mostly in the mixing layer and spent > 20 h above the sea, confirming that the conditions observed for the release experiments were captured during our campaign with high reproducibility. Only one day (21/05/2018) showed a remarkably different air trajectory for the modeled time. In that case, the air masses spent only 7 hours within the mixing layer height and travelled only above land areas.

Table 1. 24h backwards trajectories reaching the aviary during daytime during the ground campaign period. A trajectory was modeled each hour, therefore, using this approach an air mass can spend maximum 24h in the mixing layer, and depending on its coordinates it can be either above sea (marine boundary layer) or above land.

Day and time	Time mixing layer (h)	Time above sea (h)	Time above land (h)
07/05/2018 16:00	24	24	0
09/05/2018 16:00	16	12	0
11/05/2018 16:00	24	24	0
13/05/2018 16:00	24	24	0
15/05/2018 16:00	22	22	0
17/05/2018 16:00	24	24	0
19/05/2018 16:00	12	9	3
21/05/2018 16:00	7	0	7
23/05/2018 16:00	24	13	11
25/05/2018 16:00	21	21	0
27/05/2018 16:00	24	24	0
29/05/2018 16:00	12	12	0
31/05/2018 16:00	24	24	0
02/06/2018 16:00	17	17	0
04/06/2018 16:00	24	19	5
06/06/2018 16:00	24	24	0
08/06/2018 16:00	18	11	7
10/06/2018 16:00	24	24	0
12/06/2018 16:00	24	24	0

We modelled air masses forward trajectories 24h each release experiment at the time and place of release and quantified a west wind component (WWC) based on wind speed and direction as a measure of the strength of westerly winds. Indeed, most of the release experiments were conducted during westerly winds regime. An example of the type of information for a release site is given in Supplementary Fig. 6.

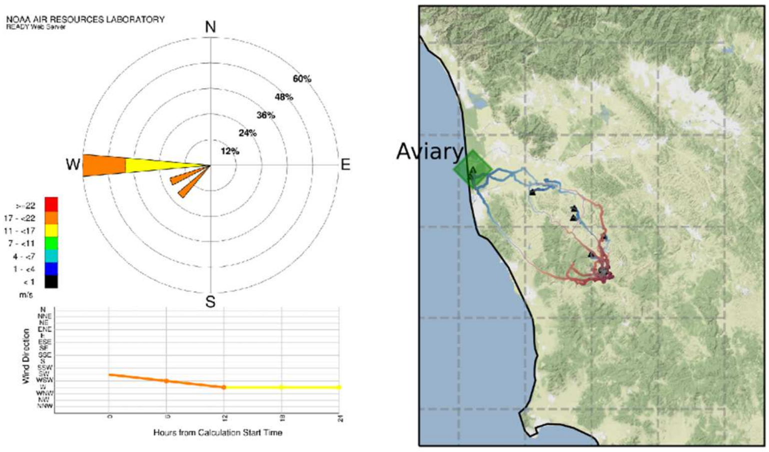

Figure 6. Wind direction and wind speed (NOAA weather archives) were modelled for the 24-h forward trajectories of the air masses reaching each release site at the time and place of each release experiment (left panels). Birds flight tracks are color coded, depending on the distance to the home loft (here indicated as Aviary) reached by each bird individual ranging from dark red for far distances to dark blue for close distances. Black markers indicate the end point of each track (last registered point by the GPS logger they bear). Bird flight tracks were drawn with iPython and combined with the NOAA READY online system archived data for the respective locations.

We calculated a westerly wind component (WWC) from the weather data extracted from the NOAA archives for the time, day and place of release of the birds release experiments. The birds' flight tracks recorded by GPS were used to extract the distance from the aviary of the last recorded point of the GPS and to calculate the mean aggregate azimuth penalty (MAAP), a measure of the direct path taken by each bird upon release and during the active flight to reach their home loft.

Mean aggregate azimuth penalty is calculated with a down-sampled version of the GPS data of each bird's flight. The flight data is sampled at hourly intervals and only these points are considered in the calculation of the mean aggregate azimuth penalty. At each sampled point in a bird's flight path, the actual heading/azimuth of the pigeon and the direct/desired heading/azimuth to reach the aviary are used to obtain the absolute azimuth penalty. The average value of the absolute azimuth penalty values over the down-sampled flight path data for a bird gives the MAAP metric.

Normalizing/averaging the sum of absolute azimuth penalty values with the number of intermediate steps allows comparing the MAAP values of different bird flights regardless of the flight duration.

The MAAP calculation procedure is depicted in Figure 7 for three examples of data. The bird flights are plotted with overlays of the bird's actual heading (in blue) and the desired heading (in green). The intermediate data points are ignored and the heading is calculated at regular time intervals only. It is observed that the birds can fly quite far in some hourly slots but at other times they do not cover as much distance.

Release location: Agliana on 2016.08.15 exp code A200

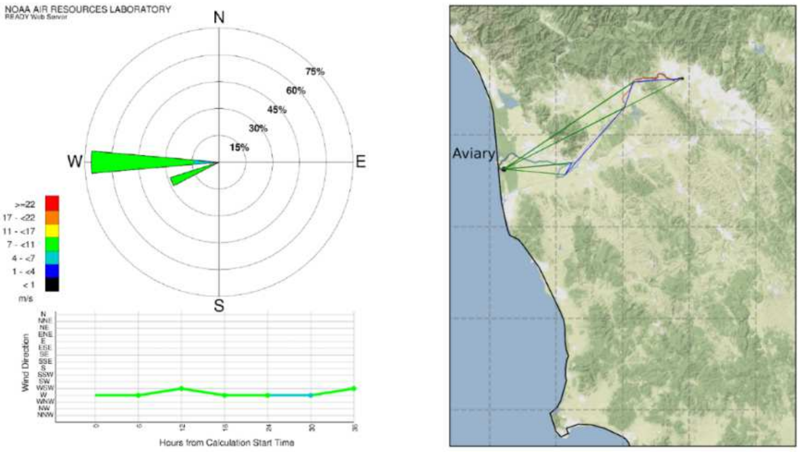

Release location: Saline on 2017.08.24 exp code A20

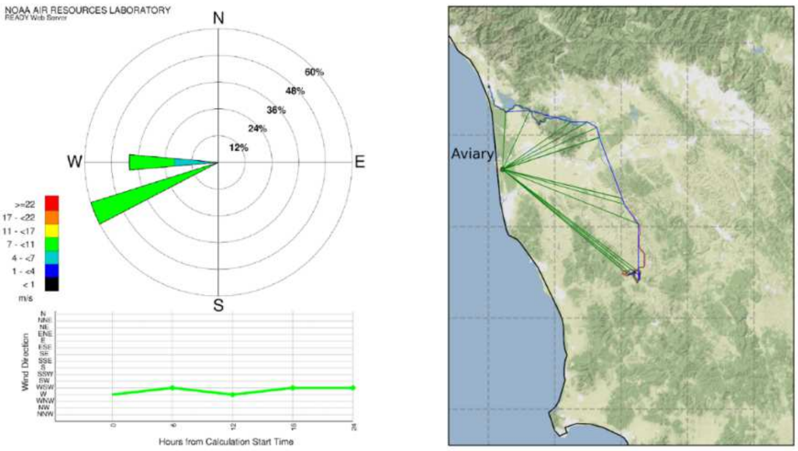

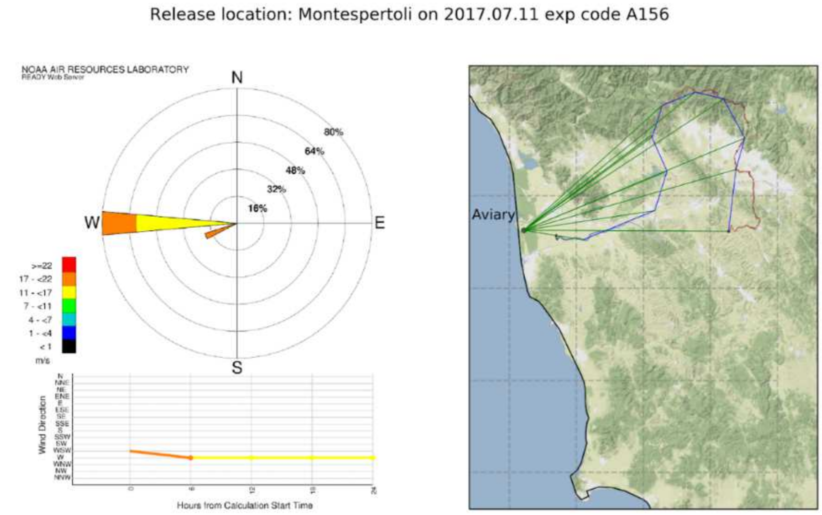

Figure 7. Wind direction and wind speed (NOAA weather archives) modelled for the 24-h forward trajectories of the air masses reaching each release site at the time and place of each release experiment (left panels). Bird flight paths with actual heading (in blue) and desired/direct heading (in green) used for calculation of the mean aggregate azimuth penalty (MAAP). Bird flight tracks were drawn with iPython and combined with the NOAA READY online system archived data for the respective locations.

The distance from the aviary and the MAAP are reported in function of the WWC in supplementary Figure 8, for each release site and each release experiment (one marker= one bird).

A general negative trend is observed in all cases, meaning that higher the WWC (=stronger westerly winds) the closer the birds approach their home (i.e. shorter distance from aviary) and the more direct their flight to home (i.e. smaller MAAP). Data were fitted through linear regression quadratic regression equations in order to find the best fitting approach.

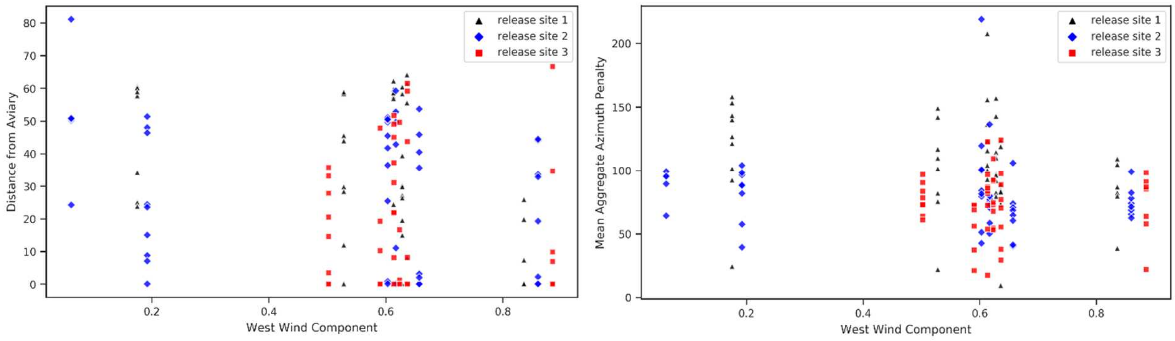

Figure 8. Distance from the aviary (left) and mean aggregate azimuth penalty (MAAP, right) are reported in function of the west wind component (WWC). Analysis of wind data were conducted from the NOAA weather archive data, from which a WWC was calculated. Distance from the aviary and MAAP were obtained from the flight tracks data of the birds release experiments. Markers indicate the different release sites. Fitting functions and statistical insights are reported in Supplementary material, Table 2.

Spearman's rank correlation coefficients and p values between the westerly wind component (WWC), mean aggregate azimuth penalty (MAAP) and distance from the aviary features are reported in Table 2.

Table 2. Spearman's rank correlation coefficients and the corresponding *p*-values between the westerly wind component (WWC), the mean aggregate azimuth penalty (MAAP) and the distance from the aviary.

	**WWC_S**	**MAAP_S**	**Distance_S**	**WWC_p**	**MAAP_p**	**Distance_p**
**WWC**	1	-0.213	-0.277	0	9.853e-03	7.207e-04
**MAAP**	-0.213	1	0.512	9.853e-03	0	4.153e-11
**Distance**	-0.277	0.512	1	7.207e-04	4.153e-11	0

Table 3. Pearson's linear regression coefficients (P), corresponding *p*-values (p) and Spearman's rank correlation coefficients (S) between the median homing efficiency index (HEI) of a group of released individuals and the time the air masses spent above land and above sea for the three release sites.

	**Above land**	**Above sea**
Release site 1	P=-0.748, p=0.002, S=-0.754	P=-0.496, p=0.04, S=-0.432
Release site 2	P=-0.809, p=0.03, S=-0.667	P=0.945, p=0.06, S=0.883
Release site 3	P=-0.584, p=0.01, S=-0.429	P=-0.606, p=0.05, S=-0.058

Figure 9 shows the aviary, the three release sites and the origin of the air masses 24-h prior the birds release time. The origin of the air masses was modeled using a back-trajectories analysis to highlight specific pollution events occurred at a specific time and location. Therefore, using the

same approach, we modeled the back-trajectories of the air masses that reached the three release sites at the time and day of the birds' release. This information is very interesting, as it can tell where the air masses have been travelling before reaching the site. It does not tell what chemical compounds are contained in an air mass, but it gives an indication of some possible pollutants present in the air mass reaching the site (for example, if an air mass traveled for 24 h in the mixing boundary layer over the sea, we can speculate that compounds emitted by the sea are contained in the air mass). The modelling was necessary as no chemical information was available from the sites at the time of the birds' releases. The colored markers therefore are placed in the position were the air masses reaching the release site originated 24h prior to the birds' release. The shape of the markers distinguishes between the three release sites: therefore, a triangular marker placed in the Alps indicates the place where the air mass reaching the release site 1 was 24h prior the release experiment took place. The color scale represents the HEI gradient for each release group (median value). We report only three back-trajectories for two simple reasons, the first is to help the reader to guide the colored markers to the release site markers, and understand that the colored markers are the origin of the air masses, the second is to make the plot less confusing.

The best homing performance from the investigated data set occurred when the air masses spent 13 h in the marine boundary layer, therefore mixing with sea's emissions. However, other good performances were achieved when the air masses originated in areas distant from the sea, therefore marine air seemed to be helpful but not essential for homing.

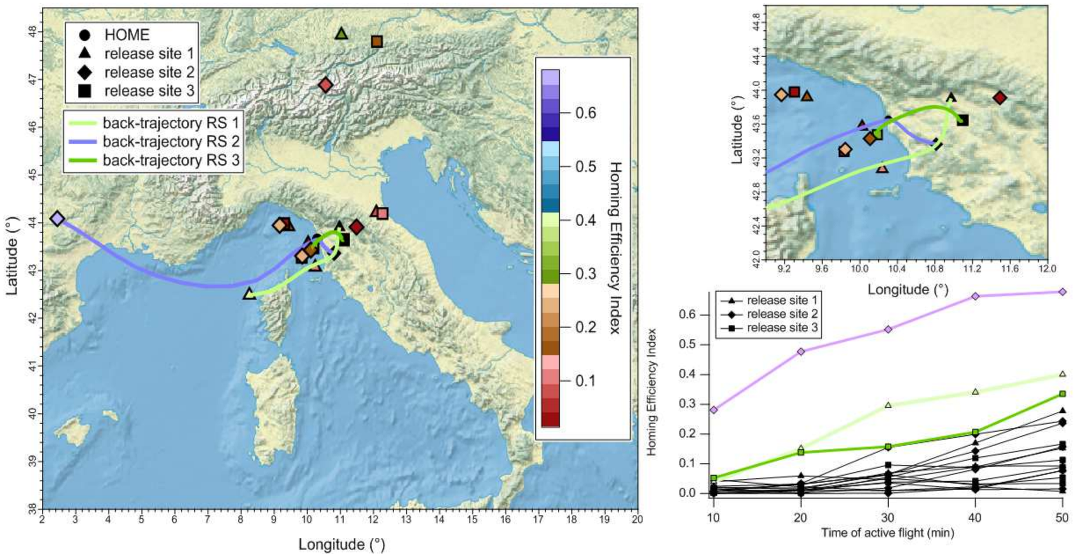

Figure 9. Origin of air masses reaching the three release sites used for the navigational experiments. The origin is modelled using HYSPLIT as 24 h prior the release time for each release experiment. The colored scale represents the homing efficiency index (HEI) magnitude, calculated as a median value of 10 individuals released in each experiment, here (left plot) reported as a function of the coordinates of the origin of the air masses. Modelled backward trajectories of the air masses are plotted for the best performance (highest HEI) achieved for each release site. Colors follow the colored scale reported in the legend. On the top right a magnification of the left figure is reported. On the bottom right, the HEI is plotted as a function of time of active flight for the group of birds released. Each line corresponds to a different experiment on a different day during summer 2016 and summer 2017. Markers refer to the release site used for the each experiment. Colors refer to the day of the reported backtrajectories on the left plot. The figure was drawn with Igor WaveMetrics.

Based on the calculated trajectories, we infer the time spent by the air masses within the boundary layer above land and sea (see method) for 24 hours prior to release and checked if this is relevant for homing. Figure 10 shows that for release site 1, there is a negative trend between HEI, computed on the section of track up to 50 min of flight, and time of the air over land. For the releases from the other two sites, a weaker but similar trend exists. When the time that air masses spent over the sea is considered, release site 2 shows a weak positive trend between HEI and time (Figure 10). These findings indicate that marine air is helpful for homing for at least one release site. It should

be noted however, that the large variability between individual performances makes this type of analysis uncertain, further studies aim at reducing this uncertainty by analyzing more flight tracks following the proposed approach.

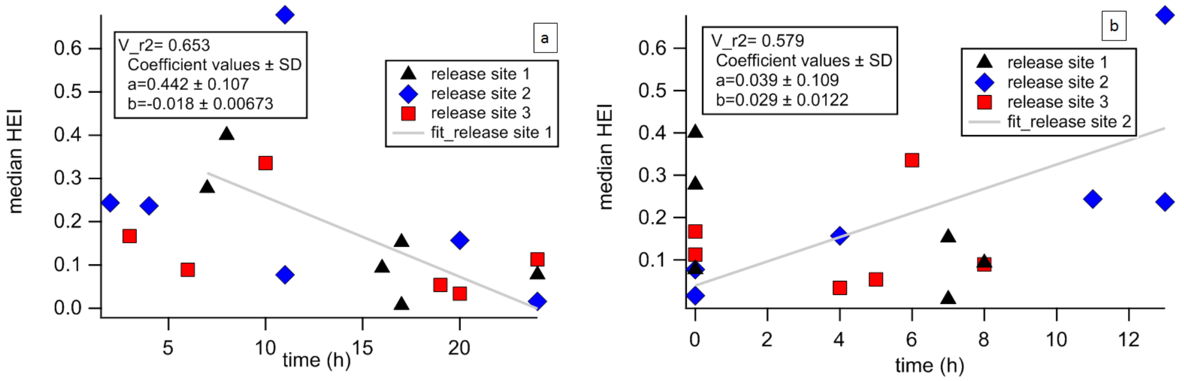

Figure 10. Homing efficiency index (HEI) median values of a group of released individuals reported as a function of the time spent by the air masses reaching the release sites above land (a) and above sea (b). Analysis were conducted with the model Hysplit, computing the backward trajectories 24 h prior the release time. Points spent by the air masses above the boundary layer height were discarded from the analysis (see method). Markers indicate the different release sites of the experiments, while linear regression coefficients reported in the panels correspond to the data points of release site 1 and release site 2, respectively. Supplementary table 3 shows Pearson's linear regression coefficients, corresponding *p*-values and Spearman's rank correlation coefficients for the three release sites.

Methods: On-line sampling

Proton transfer reaction mass spectrometry (PTR-MS) was used to monitor the composition of the air along with meteorological parameters during two months of intensive field campaign at Arnino field station (Italy). Selected protonated mass fragments and attributed identities are reported in Table 4. Table 4 shows also the limit of detection (calculated as 3 times the standard deviation of the precision) for each measured mass fragment.

Table 4. Protonated masses with attributed identity and limit of detection (LOD) monitored by PTR-MS.

Protonated Mass (m/z)	Compound	LOD 3 σ (ppt)
33	Methanol	1895
42	Acetonitrile	33
45	Acetaldehyde	83
61	Acetic acid	0.74ncps[a]
63	DMS	66
69	Isoprene	241
71	MVK [b] +MACR+ISOPOOH	58
73	MEK	93
79	Benzene	42
81	Monoterpenes[c]	67
93	Toluene	55
107	Xylene	66
121	Trimethylbenzenes[d]	51

a: The standard gas mixture does not contain acetic acid. The limit of detection of acetic acid is reported as normalized counts per second (normalized by primary ions and water cluster).
b: The standard gas only contains methyl vinyl ketone (MVK). m/z 71 was calibrated with MVK.
c: The standard gas only contains α-pinene. The fragment m/z 81 had higher signal than protonated monoterpene (m/z 137) in this study. Therefore, m/z 81 was selected to represent the sum of monoterpenes, calibrated with α-pinene.
d: The standard gas only contains 1,3,5-Trimethylbenzene.

References

Garratt, J.R., The atmospheric boundary layer. Cambridge University Press, https://doi.org/10.1002/qj.49712051919, 2006.

Lana, A., Bell, T. G., Simó, R., Vallina, S.M., Ballabrera-Poy, J., Kettle, A.J., Dachs, J., Bopp, L., Saltzman, E.S., Stefels, J., Johnson, J.E., Liss, P.S., An updated climatology of surface dimethlysulfide concentrations and emission fluxes in the global ocean, Global Biogeochem. Cycles, 25, GB1004, doi:10.1029/2010GB003850, 2011.

Yáñez-Serrano, A. M., Nölscher, A. C., Bourtsoukidis, E., Derstroff, B., Zannoni, N., Gros, V., Lanza, M., Brito, J., Noe, S. M., House, E., Hewitt, C. N., Langford, B., Nemitz, E., Behrendt, T., Williams, J., Artaxo, P., Andreae, M. O., and Kesselmeier, J.: Atmospheric mixing ratios of methyl ethyl ketone (2-butanone) in tropical, boreal, temperate and marine environments, Atmos. Chem. Phys., 16, 10965-10984, https://doi.org/10.5194/acp-16-10965-2016, 2016.
